# Uncovering viral protein acquisition events and human-specific folds with pairwise comparisons of predicted protein structures

**DOI:** 10.1093/molbev/msag110

**Published:** 2026-04-27

**Authors:** Julia C Malnak, Saira Montermoso, Frederic D Bushman, Noam Auslander

**Affiliations:** Department of Microbiology, Perelman School of Medicine, University of Pennsylvania, Philadelphia, PA 19104, USA; Program in Molecular and Cellular Oncogenesis, The Wistar Institute, Philadelphia, PA 19104, USA; Genomics and Computational Biology Graduate Group, Perelman School of Medicine, University of Pennsylvania, Philadelphia, PA 19104, USA; Department of Biochemistry and Biophysics, Perelman School of Medicine, University of Pennsylvania, Philadelphia, PA 19104, USA; Department of Microbiology, Perelman School of Medicine, University of Pennsylvania, Philadelphia, PA 19104, USA; Program in Molecular and Cellular Oncogenesis, The Wistar Institute, Philadelphia, PA 19104, USA

**Keywords:** viral protein structures, virus classification, pairwise genome comparison, viral host range

## Abstract

Pairwise sequence comparisons are at the center of molecular evolutionary analyses. However, viral pairwise comparisons are challenging because extreme mutation rates and evolutionary pressure cause genomes to diverge rapidly, limiting detectable sequence similarity to fewer than 3% of virus pairs. To overcome these limitations, we compared viruses based on structural similarity, using predicted protein structures from ColabFold and Foldseek to define protein fold clusters. We represented each virus genome by its protein structural content. Pairwise similarities between viruses were then quantified using the Jaccard index based on the presence or absence of protein fold clusters. Using a recently established viral protein fold database, we compared all pairs of eukaryotic viruses in RefSeq. This approach increased the proportion of comparable viral genome pairs from 2.4% to 16.5%. Using this protein-fold representation of viruses, we were able to accurately predict viral families with an average sensitivity of 85.9%. Investigation of viral families showing limited sensitivity with this approach uncovered a laterally transferred structural cluster (Rep/NS1) broadly shared across diverse viral families and found in the avian lineage of adenoviruses. Sequence homology suggests that this Rep was acquired from *Parvoviridae*, but the protein is mutant in the ATPase active site, indicating possible exaptation toward a purely DNA-binding function. In *Gammapapillomaviruses*, several E4 clusters were associated with human tropism. In summary, by representing viruses with structural protein clusters, we can classify highly divergent viruses, trace lateral gene transfer, and uncover features associated with viral host range.

## Introduction

Viruses are obligate intracellular parasites whose genomes represent one of the largest sources of genetic diversity on Earth. This diversity is influenced by horizontal gene transfer (Shackelton and Holmes 2004), genome segment reassortment (McDonald et al. 2016), high mutation rates (Drake et al. 1998; Sanjuán and Domingo-Calap 2016), and strong evolutionary pressures. Recent advances in metagenomics have uncovered hundreds of thousands of previously unknown viruses from diverse ecosystems (Gregory et al. 2020; Nayfach et al. 2021; Charon et al. 2022; Camargo et al. 2023). Despite these advances, taxonomic classification remains a major challenge due to the extreme diversity and mosaic nature of viral genomes. Nevertheless, classification is critical to study viral evolution and to discover the roles of viruses in diverse ecosystems globally. Cataloging viral diversity not only enables the study of virus-host interactions but also reveals processes such as gene exchange between viruses and patterns of variation that emerge within closely related viral groups. These insights have broad implications; for example, studying groups of closely related viruses improves pandemic preparedness by informing how new pathogens may emerge from global viral pools (Kuhn et al. 2019; Haltom et al. 2025). Identifying host-specific proteins or functions allows inference of host shifts, cross-species transmission events, and their potential mechanisms (Volz et al. 2013). The vast and largely untapped genetic reservoir encoded in viral genomes is a potential source of novel molecular functions with biomedical and biotechnological applications.

The classification of viruses is more challenging than the classification of cellular organisms. The tree of cellular life (Woese et al. 1990) is rooted in a last universal common ancestor and can be reconstructed from the universally conserved ribosomal RNA gene. However, viruses lack an analogous universal marker, and creation of a universal viral taxonomic structure with a single common ancestor has not been possible (Simmonds et al. 2023; Koonin et al. 2024 ). Instead, viral evolutionary histories are shaped by frequent gene acquisition, loss, and duplication events, leading to different phylogenetic trees depending on the genes chosen for reconstruction (Maddison 1997 ). Although phylogenetic trees are useful for tracing the history of viral lineages (Lefrancq et al. 2025; Pekar et al. 2025 ), they typically do not capture the extensive gene gain and loss that shape viral evolution (Sogin et al. 1993). To account for these processes, whole-genome comparison approaches have been developed, using shared proteins as indicators of similarity across viruses. These approaches typically rely on amino acid clustering to identify homologous proteins (Gao and Qi 2007 ; Cresawn et al. 2011 ; Iranzo et al. 2016). However, viruses that diverged in the distant past often lack detectable similarity even at the amino acid level, making sequence-based classification especially difficult for highly diverse groups. Incorporating structural information can help overcome this limitation by revealing broader similarities across divergent proteins. Recent advances in structure prediction now enable large-scale structure-based analysis without the need for experimental data (Caprari et al. 2015). Additionally, the development of Foldseek (van Kempen et al. 2024) allows rapid, high-throughput structural alignment. Because structure is more conserved than sequence, structural alignment has been previously used to construct evolutionary history for proteins with a high mutation rate (Moi et al. 2025). This finding demonstrates the potential for learning relationships between highly divergent sequences, which, if scaled to the whole genome, can be used to find transfer events between viruses and their hosts and uncover new functional roles of viral proteins.

Here, we developed an approach that leverages shared protein folds to quantify similarities between viral genomes, applying it to a broad analysis of phylogeny and gene flow. To this end, we used the dataset from Nomburg et al. (2024), which employed ColabFold (Mirdita et al. 2022) and Foldseek (van Kempen et al. 2024) to establish comprehensive structural clustering across all RefSeq (Goldfarb et al. 2025) eukaryotic-infecting viral proteins. Each viral genome from this dataset is represented as a vector encoding the presence or absence of these structural clusters. This approach allowed us to detect similarities across distant taxonomic boundaries that are not captured by sequence alone. With these vectors of virus structural content, we were able to classify viral genomes into their known taxonomy, recapitulating existing virus classification. Moreover, by analyzing clusters shared across diverse groups, we identified examples of lateral gene transfer between viral families, including the transfer of a Rep between *Parvoviridae* and avian-infecting *Adenoviridae*. We detected structural clusters associated with specific host ranges and taxonomic boundaries, such as the E4 proteins with structural specificity for primates. We also found that homologs for human membrane proteins with immune evasion functionality were enriched in human-infecting viruses across families. These findings highlight the potential of structure-informed approaches to reveal both evolutionary relationships and functional constraints in viruses. By integrating protein structure into comparative genomics, this study demonstrates how structural information can be used to overcome the limits of sequence-based methods and uncover fundamental principles of viral diversity and evolution.

## Results

### Structure-based comparison enhances the measurable pairwise similarity between viruses

**Figure 1:**
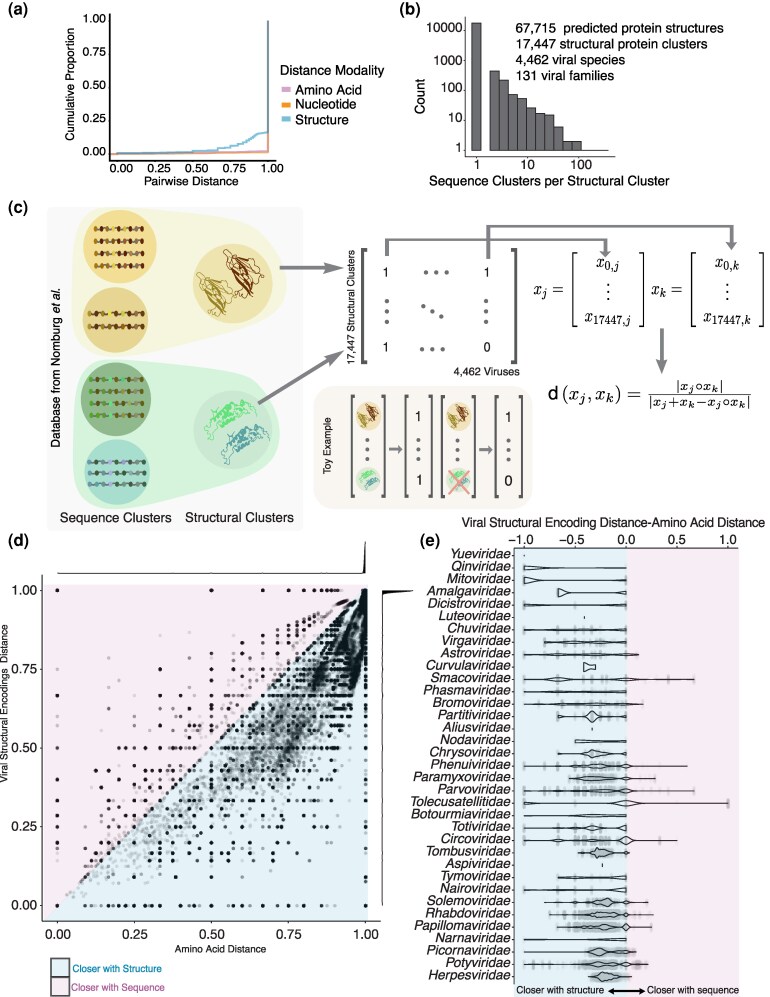
Pairwise comparisons based on protein structure increase the proportion of comparable viruses. (a) Empirical cumulative distribution of pairwise distances across all eukaryotic infecting RefSeq viruses from the Nomburg et al. dataset; nucleotide distances, amino acid distances, and viral structural encoding distances. (b) Distribution counting the number of sequence clusters (x-axis) in each structural cluster based on the Nomburg et al. dataset. (c) Workflow for establishing the viral structural encodings and evaluation of pairwise distances of viruses. Database from Nomburg et al. where each viral protein represented in RefSeq was clustered by sequence similarity. Sequence cluster representatives were folded and clustered into 1 of 17,447 structural clusters. Based on these, every virus is encoded as a binary vector with 17,447 positions, each indicating the presence or absence of each structural cluster. A distance is evaluated for each pair of viral structural encodings, x_j_ and x_k,_ using the Jaccard index. (d) Scatter plot comparing pairwise distances using amino acids (x-axis) vs viral structural encodings (y-axis). The marginal plots are density distributions of each metric. (e) Violin plot of the distribution of differences in distance metrics per viral family (y-axis). The X-axis is the viral structural encodings—amino acid distance. Each point is a pairwise comparison within the virus family. The top 35 families with the greatest average increase in structural similarity are displayed.

The objective of our study was to identify relationships between highly diverse viruses that share no detectable sequence homology. In agreement with previous studies (Iranzo et al. 2016; Mavrich and Hatfull 2017; Nomburg et al. 2024), we found virus pairs often share little to no sequence similarity. We performed all-against-all pairwise comparisons of 4,462 eukaryotic infecting viruses from RefSeq and found that fewer than 2% of genome pairs shared any quantifiable nucleotide similarity, and fewer than 3% shared similarity at the amino acid level (Fig. 1a, Figure S1a). We surmised that by grouping proteins at the structural fold level, as opposed to grouping by shared sequence similarity, we could increase the proportion of pairwise comparable viruses. This rationale is based on the observation that structure is more conserved than sequence (Illergård et al. 2009) and numerous known groups of sequence-divergent proteins have conserved folds present within viral realms, such as the double jelly roll capsid, single jelly roll capsid, RNA-dependent RNA polymerase, reverse transcriptase, rolling circle replication endonuclease, and the superfamily 3 helicases (Dimitrov 2004; Koonin et al. 2024; Nomburg et al. 2024) (Figure S1b).

To use viral protein folds for pairwise comparisons, we used a curated dataset of 67,715 ColabFold (Mirdita et al. 2022) predicted protein folds from 4,462 RefSeq eukaryotic viral species across 131 families (Nomburg et al. 2024). In the dataset, proteins were clustered into 17,447 clusters using the structural aligner Foldseek (van Kempen et al. 2024) (Fig. 1b) (Nomburg et al. 2024). Each virus is represented as a 17,447-dimensional vector, with binary values indicating the presence or absence of each structural protein cluster (Fig. 1c). These vectors are referred to as viral structural encodings. To quantify pairwise similarities for all pairs of viruses in the dataset, we computed the Jaccard index for pairs of viral structural encodings, which measures the proportions of shared protein folds relative to the number of folds present in a pair of genomes (Fig. 1c). To quantify the increase in comparable viral pairs, we also calculated the pairwise distances based on nucleotide and amino acid sequences (see Methods). The proportion of all virus pairs in the dataset with detectable similarity rises from 1.2% based on nucleotide similarity and 2.4% using amino acid similarity to 16.5% when similarity is assessed based on shared protein folds (Fig. 1a,d, Figure S1a). We also tested whether high-quality structures alone could still expand the proportion of similarities using our viral structural encodings. When removing structures with mean pLDDT below 50 or 70, 11% and 7.4% virus pairs, respectively, showed measurable non-zero similarity, still exceeding the proportion observed with sequence-based comparisons (Figure S1c). Most virus pairs show no similarity due to their diverse evolutionary origins, but the proportion of viruses with any similarity increases within the same genome type (e.g. dsDNA, dsDNA-RT, dsRNA, ssRNA). Even within the same genome type, structural encodings comparisons yield a higher proportion of comparable pairs than nucleotide or amino acid similarity (Figure S1d,e,f).

We next investigated which viral families were characterized by greater similarity when using the viral structural encodings compared to protein sequence homology. Overall, 84 (64.1%) of families showed greater average intra-familial structural similarity compared to amino acid similarity. The families with the highest gain in pairwise similarities using the viral structural encodings were *Mitoviridae*, *Qinviridae*, and *Yueviridae* (Fig. 1e). Only 4 families (3.1%) were more similar using amino acid pairwise comparisons, namely, *Alphasatellitidae*, *Bacilladnaviridae*, *Genomoviridae*, and *Hantaviridae*. The remaining 43 families (32.8%) showed no differences across 2 similarity metrics. Furthermore, we found viral structural encodings increased similarity in 19% of the between-family comparisons. The greatest increase in similarity when using structure to conduct inter-family comparisons was for *Marnaviridae* and *Dicistroviridae* (with an average of 97% points of increased similarity), both from the order *Picornavirales* (Figure S1g). Some of the families showing a gain in cross-taxonomy pairwise similarities using the viral structural encodings were characterized by minimalist genomes, such as *Nenyaviridae* and *Mitoviridae* (Figure S1g). *Mitoviridae*'s genome only encodes a diverse RdRp, not shared with any other family. However, by using structural clustering, *Mitoviridae*'s 12 sequence clusters were reduced to 2 structural clusters that are highly shared with other RNA virus families. Capsid and Rep proteins that form the genomes of *Nenyaviridae* are similarly sequence-diverse and are grouped together with structural homology. Overall, viral structural encodings expanded the comparability of viral genome pairs both at the species and at the family levels by allowing comparisons of highly divergent proteins that share a conserved structure.

### Using structural clusters as features for viral taxonomic classification

The improved pairwise comparability when using the viral structural encodings within families implies that this approach could be used for virus taxonomy classification. To explore this application, we built multi-class classifiers that use viral structural encodings to predict viral taxonomies based on k-nearest neighbor classifiers (KNN) (see Methods). We prioritized KNN as it preserves interpretability and readily accommodates the Jaccard distance metric. We implemented a KNN model with leave-one-out cross-validation to reflect the intended real-world use case of virus classification, where newly identified viruses are assigned, taxonomic labels based on similarity to previously characterized viruses.

**Figure 2:**
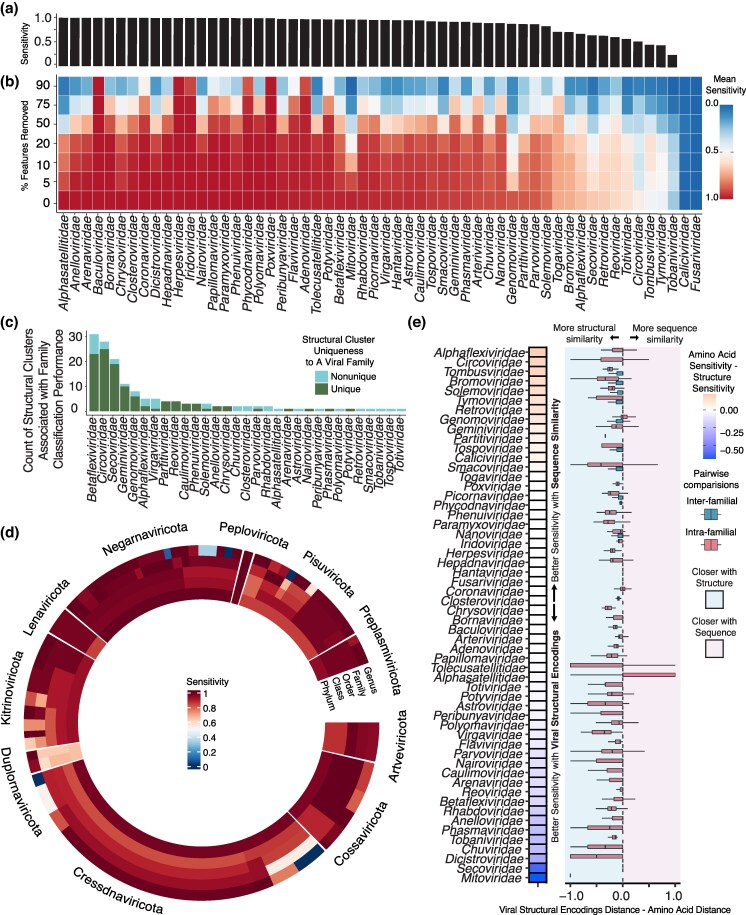
Viral structural encodings exhibit robust taxonomic classification. (a) Sensitivity of viral family classification. (b) Average sensitivity of viral family classification across bootstrap iterations of feature removal. (c) Barplot of the number of structural clusters associated with worse family classification performance. Coloring indicates whether the structural cluster is family-specific or shared. (d) Sensitivity of virus classification into different taxonomic levels. Sections in the circle are separated by the highest taxonomic level presented, the phylum. (e) Left panel: differences in sensitivity between an amino acid and structure-based virus family classifier for each virus family (y-axis). Right panel: Boxplot showing the distribution of the differences in distance metrics (x-axis) for each pairwise comparison.

Starting with family-level classification, the average sensitivity in family prediction from viral structural encoding was 85.9% (variance 0.06) (Fig. 2a). The classification sensitivity remained similar with different values for the k hyperparameter (Figures S2a, b). Classification sensitivity remained comparable when only high-confidence structures were included in the viral structural encodings. Excluding structures with a pLDDT < 50 yielded a mean accuracy of 87.5% (variance 0.03), while a pLDDT threshold of 70 yielded a mean accuracy of 80.2% (variance 0.09) (Figure S2c). The taxonomic classification was robust to bootstrapping analysis by the removal of randomly selected structural features. For example, when 20% of features were removed, the average performance dropped less than 6 points, and when 50% and 75% of the features were removed, performance decreased by an average of 19 points (Fig. 2b). Viral families with more features were more robust, and we found a positive correlation between the number of features and the mean sensitivity when 90% of the features were removed (Spearman correlation coefficient = 0.56, *P*-value < 1e-10, Figure S2d). Searching for features that consistently worsened performance when removed (see Methods), we found features that were highly specific to a viral family (Fig. 2c, Figure S2e). Families that performed poorly at baseline often contained small genomes, including *Circoviridae*. However, across all families, there was not a strong correlation between family classification sensitivity and average genome length (Spearman correlation coefficient = 0.17, *P*-value < 0.05, Figure S3a). Another observation was that these families also had a small number of features. Our rationale was that families with fewer features encoded would be harder to differentiate from other viruses that share the same features. However, the number of features in a family and its classification sensitivity were not correlated (Spearman correlation coefficient = 0.15, *P*-value = 0.27, Figure S3b). These trends suggest that factors beyond the model inputs had minimal impact on performance.

We next assessed how a classifier trained using viral structural encodings performed at different taxonomic levels. In general, the average classification sensitivity increased at higher taxonomic levels, with genus having the lowest (0.838, variance = 0.08) and phylum having the highest performance (0.914, variance = 0.01) (Fig. 2d, Figure S3c, Supplementary Dataset S1). Within each phylum, at least 1 taxonomic level achieved an average sensitivity above 0.899, indicating each virus can be accurately classified at some taxonomic level. Genera like *Cyclovirus* and *Gemykibivirus* within the phylum Cressdnaviricota were often misclassified as other members within the phylum. Their genomes are small and contain relatively few features, which tend to be broadly shared at higher taxonomic levels, as indicated by improved classification performance at those levels (Supplementary Dataset S2). Misclassification at a genus level was not limited to small genomes. Within the Negarnaviricota phylum, the *Sigmavirus* genus was always misclassified as another *Rhabdoviridae* family member from the *Vesiculovirus* genus. Consistently, viruses misclassified at a genus level are assigned to other members within the same family, and the rate of misclassification decreases at higher taxonomic levels. Together, these results indicate that all viruses share conserved structures with their taxonomic neighbors, although the taxonomic level at which structural similarity is most consistent varies across viral lineages.

Family-level classification using viral structural encodings was often comparable to classification using amino acid sequence comparisons (Figure S3d). However, we found 24 families had higher sensitivity when using structural encodings, and only 13 families performed better using protein sequences (Fig. 2e). Families with similar performances across the methods were characterized by lower diversity at a nucleotide level (Figure S3e). Some of the families that performed better using structure-based classification were families in which pairwise similarities increased when using the viral structural encodings (Fig. 2e), such as *Mitoviridae*, *Dicistroviridae*, *Chuviridae*, and *Phasmaviridae*. However, increased within-family pairwise similarities through viral structural encodings did not always indicate that those families would have improved predictions. For example, the *Bromoviridae* family performed better using sequence information for classification, even though intra-family comparisons were closer using viral structural encodings (Fig. 2e). Overall, stronger similarity within viral families naturally leads to improved taxonomic classification.

### Structural similarity across families underlies family misclassification

**Figure 3:**
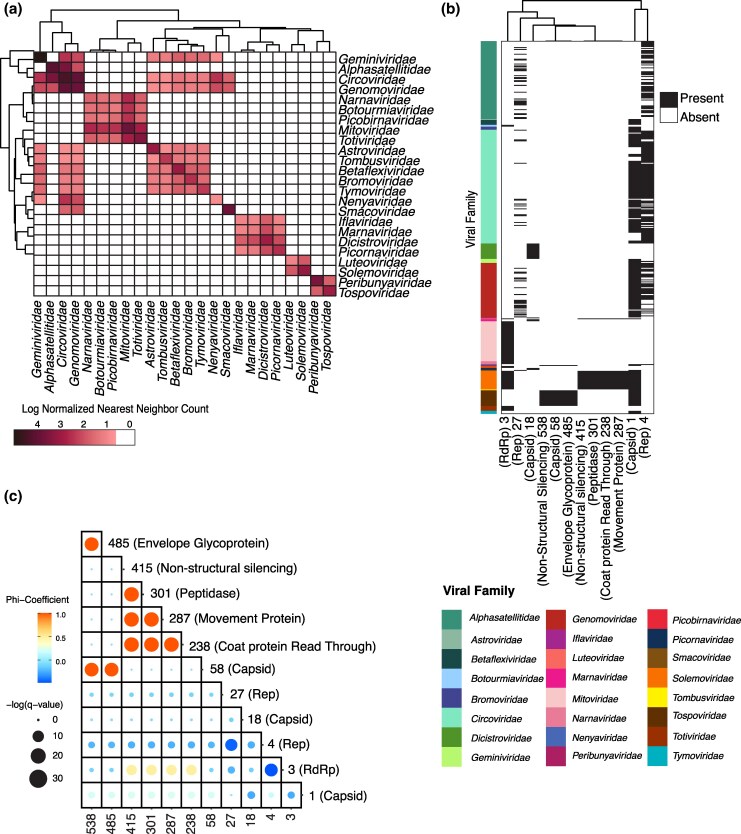
Shared structural clusters across viral families are responsible for misclassification. (a) Normalized count of within-family (diagonal) or out-of-family (non-diagonal) nearest neighbors for families that are misclassified as other families. Ties are included in the count. The count is normalized by the number of members in the family and subsequently log-transformed. (b) Structural clusters that are highly shared across viral families, and their functional description. Black indicates the presence of a structural cluster within a virus. (c) Correlation map correlating occurrences of structural clusters across viruses from panel (b), structural clusters and function. Size indicates the log-scaled FDR-corrected *P*-value, and color indicates the phi-coefficient between the 2 clusters.

To investigate cases of viral family misclassification using viral structural encodings, we explored the differences in distance between pairs of viruses within families and between families. We found that 23 out of 53 families with greater similarities using the viral structural encodings had higher classification sensitivity than sequence-based classifiers (Fig. 2e). However, 12 of these families had more intra-familial similarity with viral structural encodings but were more accurately classified using amino acid similarity. Most inter-family comparisons are narrowly centered around 0, indicating that the 2 metrics are generally similar when comparing between families (Fig. 2e). However, in cases where amino acid sequence comparisons performed better than structural encodings for virus classification, we found an increase in structural similarity to members outside of the family (Fig. 2e). Such across-family similarity can be seen in the cases of *Genomoviridae*, *Tymoviridae*, *Bromoviridae*, and *Tombusviridae*. Misclassification trends observed with the K-nearest neighbor classifiers suggest many nearest neighbors of a virus lie outside its assigned family. To investigate this trend, we found the nearest neighbor of each virus and its family classification. Twenty-four viral families contained at least one member whose nearest neighbor was from a different family (Fig. 3a). Additionally, there were clusters of viral families that were each other's nearest neighbors. Members of the Orthornavirae kingdom, such as viruses within *Picornaviridae*, *Dicistroviridae*, *Marnaviridae*, and *Iflaviridae* families, exhibited high similarity, even though virus structural encodings rarely led to misclassification (Figure S4a). *Geminiviridae*, *Circoviridae*, and *Genomoviridae* families were often misclassified at the family level (Figure S4a). These families formed a cluster with homogenous structural encodings (Figs 3a,b), indicating high functional sharing among them. To investigate clusters driving misclassification across families, we examined viruses frequently assigned to other families and found many shared functions (Fig. 3b). Highly shared clusters included members annotated with core viral functions, such as capsid or RNA-dependent RNA polymerase (RdRp). There was some heterogeneity within families, where a specific function was represented by multiple distinct structural clusters. While typically only 1 of these structural clusters would be shared across families, in some cases, multiple clusters consisting of the same function were shared across families. For example, Rep from cluster 4 is mutually exclusive with Rep from cluster 27 (Fig. 3c), yet both were shared across multiple viral families (Fig. 3b). Some structural clusters also co-occurred across families, such as clusters 238, 287, 301, and 415, which represent non-structural proteins found in *Solemoviridae* and *Luteoviridae* (Figs 3b,c). Overall, the most shared clusters among families were restricted to within viral realms, the highest taxonomic rank defined by the International Committee on Taxonomy of Viruses (Gorbalenya et al. 2020), with clusters containing proteins with canonical and highly conserved functions such as capsid and Rep, which were shared across realms (Koonin et al. 2024; Nomburg et al. 2024).

Some apparent cross-family similarities are due to incorrect or outdated annotations. Ixeridium yellow mottle virus 1, previously labeled as *Luteoviridae*, has been recently corrected to be classified as *Solemoviridae* by NCBI, in agreement with our model. Another case, the Mulberry banding virus, labeled as *Peribunyaviridae*, was classified by our model as *Tospoviridae*. We found that its RdRp aligns most closely to *Tospoviridae* (Figure S4b), supporting the classifier's assignment.

### Viral structural encodings reveal widespread Rep protein capture in *Aviadenovirus*

**Figure 4:**
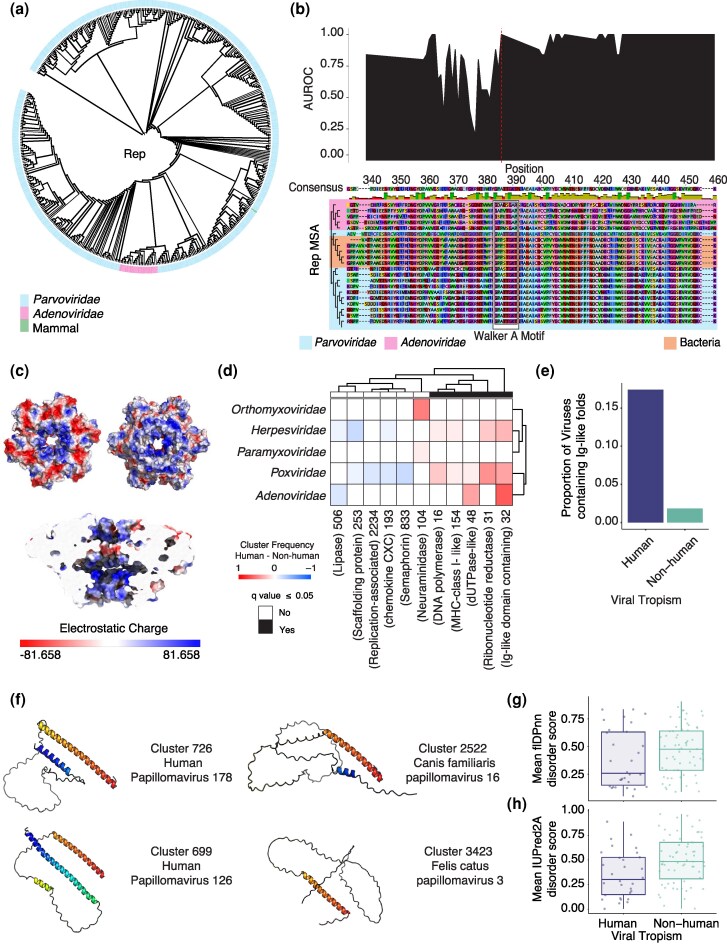
Viral structural encodings unravel horizontal gene transfer events and proteins with host specificity. (a) Phylogenetic tree of widespread Rep proteins in *Parvoviridae* (blue) and *Adenoviridae* (pink). Integration event of a Rep protein into genomic Elephant DNA is also noted in green. Tree branch width indicates bootstrap value. (b) Multiple sequence alignment positions are indicated, aligned with AUROC of the sequence model when predicting the origin of a Rep sequence (*Parvoviridae* or *Adenoviridae*). The red line indicating AUROC = 1 in the Walker A motif is in position 386. Bacterial Rep proteins are included to demonstrate the conservation of the Walker A motif across taxonomic boundaries. (c) Electrostatic potential is displayed on the predicted *Aviadenovirus* Rep hexamer, with red indicating negative charge and blue indicating positive charge. The top panel displays the exit (left) and entry (right) region of the nucleotide pore. The bottom panel is a view of the pore's channel. (d) Structural clusters are observed more frequently in human-infecting (red maps) or nonhuman-infecting (blue maps) viruses. Significantly enriched clusters are marked. (e) The proportion of viruses with a specific tropism containing 1 or more structural clusters with an immunoglobulin domain annotation. (f) E4 representatives from human (left) and non-human infecting (right) *Papillomaviridae*. Rainbow coloring reflects the protein region. The N-terminal region is colored in blue, and oranges/reds indicate the C-terminal region. (g,h) Mean intrinsic disorder scores (y-axis) predicted by flDPnn (g) and IUPred2A (h) for the first 25 amino acids in E4. Scores range from 0 to 1, where values closer to 1 indicate likely intrinsic disorder and scores of 0 indicate a stable conformation.

Many clusters comprise proteins with functions that are widespread across diverse viral groups. This pattern suggests horizontal gene transfer, as viruses from different realms have distinct ancestors and thus could not have inherited these functions by descent (Koonin et al. 2020). One key shared cluster is cluster 4, which contains the canonical Rep protein required for DNA replication in small single-stranded DNA viruses (Koonin et al. 2020; Desingu and Nagarajan 2022). Unexpectedly, this cluster was also present in viral families such as *Poxviridae* and *Adenoviridae* (Figure S4c), which are not known to use rep-mediated rolling circle replication. Within cluster 4, some members contained the ATPase and oligomerization domain, which constitutes a superfamily 3 helicase but lacked the linked HUH endonuclease domain (Figure S4d). This fold is widespread and conserved in *Aviadenoviruses*, an avian lineage of adenovirus and closely related to avian lineages of *Parvoviridae* (Fig. 4a). This *Aviadenovirus* Rep showed distant sequence homology to Parvoviral Rep40, involved in DNA packaging, and NS1 proteins in bacterial lineages (Fig. 4b). Additionally, there was distant sequence homology to a eukaryotic homolog, which has been previously characterized as a parvovirus integration event into the genomic DNA of elephant (Kobayashi et al. 2019) (Fig. 4a). The *Aviadenovirus* protein sequence also shared sequence homology with the conserved domain of parvovirus NS1, which is annotated as having ATPase function.

*Aviadenovirus*es encode an ATPase/helicase function through their Iva2, a core protein of *Adenoviridae*, initially suggesting a duplicate function of the acquired Rep homolog (Ostapchuk and Hearing 2008) (Figure S4e). To investigate the possible function of this cluster, we searched for conserved regions in these proteins, distinguishing between the *Parvoviridae* and *Adenoviridae* rep. We constructed a Long Short-Term Memory model to classify windows of protein sequences to identify regions distinguishing *Parvoviridae* from *Adenoviridae* Rep proteins (see Methods). One region that was conserved within each clade but distinguished between families was the Walker A motif (Fig. 4b). The motif is well-conserved in *Parvoviridae* and bacteria but diverged in *Aviadenovirus*, and thus the classifier could perfectly distinguish between the 2 families. The canonical Walker A motif GXXXXGK[ST] is present in Bacteria and *Parvoviridae*, but the *Aviadenovirus* consensus sequence differs: “GXXXXGA[PR]”. The loss of the threonine and lysine in *Aviadenoviruses*, which is necessary for coordinating phosphate binding (Walker et al. 1982; delToro et al. 2016), suggests that this protein can no longer bind ATP. In phage λ, mutations of lysine to alanine reduce but still retain helicase activity (Tsay et al. 2009). Although deviant substitutions are functional, the proline substitution is deleterious to ATPase function (delToro et al. 2016). Because this protein is located near the replication machinery, we investigated whether it might contribute to replication by binding DNA (Figure S4e). Estimating the protein's electrostatic potential, we observed a positively charged channel in the modeled Rep hexamer structure, which indicates the protein retained DNA binding potential (Fig. 4c). While knock-out assays showed this protein was not required for Fadv-4 replication in chicken hepatocellular carcinoma cells (Pan et al. 2021), involvement of this protein in replication is possible for other cell types or physiological contexts.

### Viral structural encodings reveal human-tropic clusters and pinpoint the structural specificity of *Gammapapillomavirus* E4 proteins

We investigated whether there were conserved viral folds that are associated with human host specificity. By annotating viruses with known human-tropism, we identified structural clusters that were more prevalent in human-infecting viruses than in non-human infecting viruses across multiple families (Fig. 4d). We identified 2 clusters enriched in human-trophic viruses, both containing Ig-like folds: cluster 32 (mean pLDDT = 71.6) and cluster 154 (mean pLDDT = 74.7). For example, cluster 32, a membrane protein containing an immunoglobulin domain, was strongly enriched in human viruses, particularly in human-infecting *Adenoviridae*. Many proteins in this cluster are homologs to human-encoded surface proteins and are known to be horizontally transferred (Farré et al. 2017). Despite this shared characteristic, functional mechanisms of proteins in this cluster vary across viral families. For example, in *Adenoviridae,* E3 CR1-β is a membrane protein that binds CD45 on leukocytes, inhibiting NK and CD4 T cell activation (Windheim et al. 2023). In *Herpesviridae*, the fold is widespread and diversified (Soh et al. 2024). One example of these folds in *Herpesviridae* is BARF1, an oncogene encoded by Epstein–Barr virus (EBV), which is a CD80 homolog (Tarbouriech et al. 2006) that binds to macrophage colony-stimulatory factor, blocking macrophage differentiation and activation (Strockbine et al. 1998; Hoebe et al. 2012). Another enriched cluster, 154, contains human-associated membrane proteins from *Poxviridae* and *Herpesviridae*. These are homologs of MHC-I-like proteins. In *Poxviridae*, the 2L protein lacks a peptide-binding groove and β2-microglobulin light chains (Yang et al. 2009) but instead binds the tumor necrosis factor TNF to prevent downstream immune signaling and apoptosis (Brunetti et al. 2003). In *Herpesviridae*, the MHC-class 1 homolog UL18 serves a similar function by binding LIR-1, an NK inhibitory receptor (Chapman et al. 1999; Yang and Bjorkman 2008). Many human-enriched structural clusters shared across viral families were homologous to human receptors, reflecting viral adaptations to modulate host immunity (Lasso et al. 2021). We examined non-human viruses containing Ig-like folds from clusters 32 and 154 and found they were all animal-infecting viruses (Figure S5a), suggesting that the fold may have originated in animal-infecting viruses, and the presence in human-infecting viruses could result from zoonotic spillover. In contrast, we found no evidence of broader primate-specificity. Repeating the enrichment analysis in primate vs non-primate infecting viruses showed only a marginal increase in enrichment relative to the human-specific enrichment score (Figure S5b).

Ig-like folds occur in multiple structural clusters. Therefore, we tested whether their enrichment in human-tropic viruses was general or cluster-specific. Clusters were annotated as containing Ig-like folds if any member was annotated with an immunoglobulin domain through Interproscan (Jones et al. 2014). Overall, Ig-like folds were more common in human-infecting viruses (18.6%) compared to non-human infecting viruses (1.8%, Fisher’s exact test *P*-value: <1 × 10^−10^) (Fig. 4e). Most Ig-like folds were in *Adenoviridae*, *Herpesviridae*, *Flaviviridae*, and *Poxviridae*, but only *Adenoviridae* and *Poxviridae* showed a significant association with human tropism (Figure S5c Fisher’s exact, *P*-value = 0.001 and 0.03 for *Adenoviridae* and *Poxviridae*, respectively). Many other Ig-fold containing clusters were family-specific, often to *Poxviridae*. Ig-like fold clusters generally did not merge due to differences in length and prediction quality (Figure S5d-f).

We next investigated human-specific structural clusters characteristic of specific viral families. To this end, we trained a KNN classifier to predict human versus non-human infecting viruses within each family using the viral structural encodings (see Methods). The classifier achieved reliable performance (AUROC > 0.7) in predicting human-infecting viruses within *Papillomaviridae*, *Picornaviridae*, and *Anelloviridae* families (see Methods, Figure S6a, b). In *Picornaviridae*, there were some structures specific to viruses with human hosts. However, these clusters were not prevalent among human-infecting viruses (Figure S6c). In *Anelloviridae*, 2 clusters of unknown function (cluster 541, 1310), which are present across multiple viruses in the *Gammatorquevirus* genus, contributed to human host specificity prediction (Figure S6d).

The strongest association was found in *Papillomaviridae*, where we identified multiple E4 structural clusters with primate-specificity, all within the *Gammapapillomavirus* genus. E4 protein is highly expressed during viral replication. The precise function of E4 is unknown, but functional studies point to E4 involvement in G2 disruption (Knight et al. 2004) and in binding keratin intermediate filaments (Roberts et al. 2003). E4 fold clusters 699 (representative pLDDT = 68.44) and 726 (representative pLDDT = 65.54) were abundant in viruses that were accurately classified as human-infecting (Figure S6e). The structural representative of these protein clusters contains N-terminal folds, while E4 representatives from non-human-infecting viruses appear to have an intrinsically disordered N-terminus (non-human infecting representatives pLDDT = 62.72, 65.97) (Fig. 4f, Figure S6f). Across all clusters, N-terminal folding is more prevalent in human-infecting viruses but is not unique to human tropism (Figure S6g). We find that all predicted N-terminal folds across *Gammapapillomaviruses,* except one, had a pLDDT > 70 (Figure S6g), supporting the interpretation that these regions are more likely to represent folded domains rather than intrinsically disordered regions incorrectly predicted as spurious alpha helices (Alderson et al. 2023). Across all clusters, human-infecting clusters had more members (Figure S7a), which may have improved prediction quality by increasing the number of protein sequences in the initial multiple sequence alignment used for structure prediction (Figure S7b).

To further evaluate the implied association between N-terminal folding and tropism using an independent approach, we predicted intrinsic disorder in N-terminal regions of all E4 protein sequences in the dataset. For each sequence, residue-level disorder was quantified using two recently benchmarked intrinsic disorder predictors (Kurgan et al. 2023): IUPred2A (Mészáros et al. 2018) and flDPnn (Hu et al. 2021) (Figure S7c,d). We then averaged disorder scores across the first 25 N-terminal residues. Both methods predicted that E4 proteins from human-infecting viruses are less disordered in the N-terminal regions from non-human infecting viruses (2-sided Wilcoxon-rank sum test, *P*-value = 0.007 and 0.08, for IUPred2A and flDPnn, respectively, Figure G, H). Functional studies characterized the role of the N-terminal region in keratin association, where loss of this domain in *Mu* and *Alpha* papillomaviruses has been associated with loss of the keratin binding phenotype (Doorbar 2013).

We found that 5 *Gammapapillomaviruses* represented in the dataset did not have an annotated E4 protein. We annotated 4 putative E4s (see Methods) with distant homology to other E4s (Supplementary Dataset S3). Three of these E4s also contained characteristic domains of the E4 protein (Figure S7e). We modeled the 3 E4s with AlphaFold3 (Abramson et al. 2024). Two of these had folds in their N-terminal region, while the E4 from HPV 184 was intrinsically disordered (Figure S7f).

## Discussion

This study proposes viral structural encodings as a genome-level representation, capturing protein structural content to enable cross-virus comparisons. Viral structural encodings increase the number of comparable virus pairs seven-fold compared to protein sequence-based comparisons. Viral structural encodings allow classification of viruses at different taxonomic levels, providing an interpretable framework to investigate shared features across viral taxonomies. For example, we were able to find cases of horizontal gene transfer between bacteria, *Parvoviridae*, and *Adenoviridae*. In addition, we demonstrate that viral structural encodings can be used to identify features associated with viral tropism. We found human-enriched folds across viral families that were remote homologs to human proteins involved in molecular recognition and immune system interactions. By associating protein structures with tropism within families, we identified human-trophic E4 clusters in *Gammapapillomavirus* characterized by predicted N-terminal alpha helices, whereas non-human E4s show greater intrinsic disorder in this region.

The advent of studies performing virus discovery and categorization from high-throughput metagenomic sequence data relies on the accurate classification of viruses based on sequence (Koonin et al. 2020; Zerbini et al. 2023). A challenge is that new viral sequences are commonly highly divergent and may not align to viruses that have been previously discovered (Bin Jang et al. 2019; Zielezinski et al. 2025). We show that sequence-diverse viruses are more measurably similar using structure-based comparisons compared to sequence-based comparisons, potentially allowing improved classification. Taxonomic classifications using viral structural encodings were robust to the removal of half or more of the features (Fig. 2a), suggesting a potential utility in metagenomics where assembled viral contigs are often incomplete. In metagenomic sequencing, samples are commonly dominated by prokaryotic viruses (Gregory et al. 2020; Camargo et al. 2023), where taxonomy is complicated (Turner et al. 2023) by the vast diversity of phage sequences (Turner et al. 2024). Since the taxonomic hierarchy of prokaryotic viruses is complex, patterns of shared structures may illuminate the origins and evolution of new phage sequences (Nasir and Caetano-Anollés 2015).

Our framework can allow the construction of protein fold-sharing networks to depict horizontal gene transfer events and evolutionary dynamics in viruses infecting eukaryotes (see Methods, Figures S4c, S8a,b). Future development of this approach will allow a similar framework for exploring shared folds in phages by accommodating the increase in diversity and number of prokaryotic viral protein fold clusters (Bouras et al. 2025). Taxonomic classification with structural features could be improved with more sophisticated model architectures, but this would reduce the inherent interpretability of classifications based on the most similar viral members. One potential improvement within the KNN framework is to weigh classification votes by distance, which would enhance classification sensitivity. Beyond classification, our framework enables linking viral structural features to phenotypes such as host tropism, demonstrated here, as well as disease association or response to antiviral therapeutics in the future.

The current study is constrained to proteins within eukaryotic virus RefSeq, excluding databases of draft viruses, which may be contaminated with nonviral proteins (Breitwieser et al. 2019; Ge et al. 2025). Because of this limitation, not all viral proteins are captured in the structural encodings. Additionally, in some viral families, classification sensitivity is limited when no structural similarity exists with other family members, as illustrated by *Retroviridae* (Fig. 2e). Yet, in most cases, misclassifications at a lower taxonomic level can be mitigated by assigning viruses to a higher taxonomic rank (Fig. 2d). Another consideration for applying this framework is the reduced performance on small genomes, most of which were harder to compare due to their minimalist content, as exemplified by *Cressdnaviricota* viruses (<6 kb) (Rosario et al. 2012). Yet, some small-genome viruses such as *Anelloviridae* (1.6-3.9 kb) (De Koch et al. 2025) were accurately classified using viral structural encodings and exhibited many family-specific clusters (Fig. 2c). This framework is specifically useful for comparing highly divergent viruses within and across taxonomic levels.

This study shows that broad structural homology can unify comparisons across highly diverse viral populations. The detection of lateral gene transfers and phenotype-specific structures underscores the potential of this framework to illuminate viral evolution and host adaptation.

## Materials and methods

### Data sources

The Nomburg et al. dataset comprised of all eukaryotic-infecting viruses with proteins represented in RefSeq in July 2022 and clustered by shared protein folds as described in ref (Nomburg et al. 2024). The Nomburg et al. structural clusters dataset and intermediate files were downloaded from https://zenodo.org/records/11156521 on 13 December 2024. Nucleotide accessions for 4,492 sequences representing 3,041 eukaryotic-infecting viruses were obtained from the accompanying ncbi_genome_lengths.csv file (Supplementary Dataset S4). Protein accessions of sequences that were clustered into structural clusters were obtained from the merged_clusters.tax.tsv file (Supplementary Dataset S5). The protein accessions represented proteins in 4,462 viral species. The number of proteins in the dataset is reported to be 67,715 proteins in the original manuscript (Nomburg et al. 2024), but after deduplicating IDs, the number of proteins in the dataset is 67,521. All sequences were downloaded from NCBI using the Bioconda installation of the Entrez Direct tool v24.0 (he881be0_0).

### Computing pairwise nucleotide similarity between viral genomes

Nucleotide pairwise comparisons of 4,492 nucleotide sequences resulted in 10,086,786 total viral genome pairs. Distances between all pairs were quantified using MASH (version 2.3) with default parameters (k = 16, s = 1000) (Ondov et al. 2016). For 564 of the 3,041 viral species, there were multiple reference genomes. For these genomes, the viral pairwise distance was averaged across all references (Supplemental Dataset S3). To compute the within-family diversity, family labels were acquired from merged_clusters.tax.tsv file, and the distance across all family members was used to compute within-family diversity.

### Computing pairwise amino-acid similarity between viral genomes

67,715 protein sequences represented in the Nomburg et al. dataset were downloaded and clustered using MMSeqs2 (Hauser et al. 2016) (release version 93d74798d7fc584e28ca35ef9522dee7d9747c82) with a minimum sequence identity set to 20%, coverage to 70%, yielding 21,837 sequence clusters. This allowed the construction of a 21,837×4,639 matrix, where rows correspond to protein sequence clusters, and columns to viruses. An element (i, j) in the matrix was denoted as 0 or 1, indicating whether a protein from species j was contained in the i^th^ cluster. The column names were matched between the structural cluster and sequence cluster matrices, testing the same set of viruses across all benchmark and metric comparisons, which filtered out 177 species without taxonomic IDs in the sequence matrix.

### Defining viral structural encodings for calculation of amino acid distance and evaluation of pairwise similarity

The complete Nomburg et al. dataset contains 18,192 structural clusters represented in 4,462 viruses across 131 families. We removed singleton clusters if the species also did not have a taxonomic ID, yielding 17,447 structural clusters. We then constructed a binary matrix of shape 17,447×4,462, where rows correspond to structural clusters and columns correspond to viruses. Each element (i, j) of the matrix is represented as 0 or 1, indicating the absence or presence, respectively, of a given cluster i within a species j as determined from the merged_clusters_counts.tsv file. We consider the columns from this matrix, i.e. the vector of presence or absence of a structural cluster in a viral species, as viral structural encodings.

To infer whether protein structure confidence impacted our results, viral structural encodings were modified so that low-quality structures were treated as absent (encoded as 0). Confidence thresholds 50 and 70 were chosen based on the Alphafold pLDDT scale (Abramson et al. 2024).

### Comparing nucleotide, amino-acid, and structural encoding distances across virus pairs

To compute virus pairwise similarities using either the viral structural encodings or the sequence cluster matrix, we computed a Jaccard index between all column pairs, representing pairs of viruses, resulting in 9,952,491 pairwise comparisons. For the representation matrix as M, the Jaccard index over a pair of viruses < J,K > is defined as $J_{J,K}=\frac{M_{J}\cap M_{K}}{M_{J}\cup M_{K}}$. The distance is defined to be 1- $J_{J,K}$. These similarities and distances were computed for all pairs of viruses. For computing nucleotide similarity, 564 of the 3,041 viral species had multiple reference genomes, the mash distance between pairs was averaged across all reference genomes. We computed the proportion of similar virus pairs by dividing the number of non-one distance pairs by the total number of pairs.

To investigate whether virus pairs were closer using structure, we computed the difference in similarity metrics between viral structural encoding distance and amino acid distance for each pairwise comparison. Negative values denote virus pairs that are more similar using the structural encodings, whereas positive values indicate pairs that are more similar using amino acid sequences. The families were ranked, in descending order, by the mean difference in similarity metrics. The top 35 families were selected for visualization in Fig. 1f.

### Taxonomic classification of viruses using KNN models

Taxonomic classification was performed using a K-nearest neighbors (KNN) model using the scikit-learn package (1.5.2). For consistency, the Jaccard distance on the viral structural encodings was used as the metric to determine nearest neighbors in the KNN classifier. For family classification, the number of members across all virus families was used to determine possible values for hyperparameter *k* (Figure S9a). K values of 3, 5, and 11 were evaluated because they were hyperparameters below the median number (14.5) of members in each family. We also confirmed that the selection of k would not be biased toward viruses with different molecule types (Figure S9b). We found that only 3 out of 13 groups of viruses had a median number of members across families that fell below 11, but the top 45% of all groups had viral families with 11 or more members. The increase of the hyperparameter k marginally improved model performance, but the observed improvement was not significant (Figure S9c, linear mixed effects model, likelihood ratio test, *P*-value = NS). A k of 11 was used for classification of all taxonomic levels because it had the highest average family classification performance and still included most taxa within each taxonomic level (Figures S9d, e, f, g)

For KNN models evaluated, viral taxonomies with fewer than k-1 (10) members or those lacking specific taxa annotations were ineligible for classification. To test the performance of the KNN model, each species was iteratively removed and classified using the remaining points (analogous to leave-one-out cross-validation). We defined taxa performance as sensitivity, or $\frac{\mathrm{TP}}{\mathrm{TP}+\mathrm{FN}}$ for viruses within each specific taxonomic level considered.

To benchmark the performance of this approach when using the viral structural encodings compared to amino acid sequence clusters, we trained another family KNN classifier using the same hyperparameters, distance metrics, and evaluation framework, but using the binary indication of the presence of amino acid clusters to represent viruses as model input. The differences in performance between the protein sequence-based and viral structural encoding-based classification were evaluated using a 2-sided paired *t*-test.

### Robustness analysis of taxonomic classification via bootstrapped feature removal

The robustness of the KNN classifier to missing features was measured by randomly removing subsets of features and using the remaining features for classifying viral families using the reduced viral structural encodings. We randomly removed 5, 10, 20, 50, 75, and 90% of features and reclassified viral species, using the leave-one-out strategy detailed above. Sensitivity was calculated for each family based on the proportion of correctly classified members when each was removed and reclassified individually. To obtain a robust sensitivity estimate, we averaged family sensitivity across 100 iterations of this procedure.

### Post-hoc feature ablation analysis

Family-specific feature importance was estimated using the results from the robustness analysis (as described above). There were six batches of feature removal, each with a different percentage of features selected for removal (5, 10, 25, 50, 75, 90). There were 100 iterations in each removal batch, and for each iteration, the family sensitivity and removed features were reported. Within each of the removal batches, we designated features as important if the family sensitivity decreased from the sensitivity using the full feature set in ≥ 50% of iterations where the structural cluster was removed. To reduce spurious associations, features were retained only if $O\geq \;\frac{E[O]}{2}$, where O is the number of times a feature is removed, and E[O]=100p where *P* is the proportion of features removed in that batch. After this filter, we further investigated features that met these criteria in all removal batches.

Additionally, some features could be associated with lower family classification performance. We evaluated which structural clusters reduced performance using the criteria that the family sensitivity increased in ≥50% of iterations where the structural cluster was removed. We applied the same filter as described above by selecting features that satisfied $O\geq \;\frac{E[O]}{2}$. Additionally, since none of the families had improved sensitivity when ≥ 50% of features were removed, features were designated as unfavorable for classification they were selected in the 5, 10, and 25% removal batches.

### Phylogenetic analysis for *aviadenovirus* rep proteins

The ORF2 from Pidgeon adenovirus (YP_009047088) was aligned against the conserved domain database using CD-search (Marchler-Bauer and Bryant 2004). We used PSI-BLAST (Altschul et al. 1997) using the ORF2 sequence as a query for 4 iterations, filtering out hits that were metagenomic assembled genomes. The resulting PSI-BLAST hits were aligned using MAFFT v7.490 (Katoh et al. 2002), and the resulting multiple sequence alignment was used to build a phylogenetic tree using FastTree 2.2 (Price et al. 2009). Trees were visualized using iTOL (Letunic and Bork 2021). To investigate the distant homology of the Rep proteins to other organisms, a PSI-BLAST (Altschul et al. 1997) was performed with Rep40 (YP_680424.1) over 3 iterations. The multiple sequence alignment was constructed, manually pruned for sequences that did not align, and visualized in Geneious Prime 2025.2.1.

### Sequence model for determining discriminative mutated regions in the *aviadenovirus* rep

*Parvoviridae* and *Adenoviridae* proteins were retrieved from PSI-BLAST searches for 3 iterations using Rep40 as query (YP_680424.1) and used for multiple sequence alignment (MSA) (Altschul et al. 1997) with MAFFT v7.490 (Katoh et al. 2002). Alignment amino acid and gaps were embedded using a linear layer to map the alphabet size of 21 to a vector of 64. The embedding was passed into 1 LTSM unit with 128 hidden states, with a subsequent feed-forward classifier composed of 2 layers, 1 with ReLU activation, and another with a sigmoid activation for binary classification of the sequence. The model was trained with PyTorch 2.6.0. using a mini batch size of 16 with 10 epochs and the Adam optimizer, defining Binary Cross Entropy loss for training. The sequence model was trained to distinguish *Parvoviridae* from *Adenoviridae* and evaluated using a sliding MSA window of size 6, including only windows with fewer than a third gap position across the alignment. Two-thirds of sequences used for training (n = 20, 10 *Parvoviridae* and 10 *Adenoviridae)*, one-third used for validation (n = 10, 5 *Parvoviridae* and 5 *Adenoviridae*). The AUROC of the validation set was calculated for each window to determine conserved regions distinguishing *Parvoviridae* from *Adenoviridae*.

### Modeling the *aviadenovirus* rep hexamer

Initially, a predicted atomic model of the bird adenovirus Rep (1–270) monomer was generated using the Alphafold3 program implemented in the Alphafold Server (Abramson et al. 2024). Structural comparison of the predicted Alphafold3 model revealed high similarity to AAV2 Rep40 and AAV2 Rep68 helicase domains. To gain insights into the potential DNA-binding capability of the bird adenovirus Rep, we generated a hexameric model based on superposition with hexameric AAV2 Rep68 helicase domain in complex with ssDNA and ATPγS atomic model (PDB:7JSI) and model fitting into the corresponding cryo-EM map of Rep68 hexamer (EMD-22455) (Santosh et al. 2020). First, we split the Alphafold3 model of bird adenovirus Rep into 2 fragments: 1-70 amino acids, which form the putative oligomerization domain, and 71-270 amino acids, which form the putative ATPase domain. We then aligned the fragments into the Rep68 using the matchmaker tool in UCSF ChimeraX v1.9 (Meng et al. 2023), connected the 2 fragments, and adjusted the atomic model to fit the Rep68 hexameric cryo-EM map using real-space refinement in Coot v0.9.8.96 EL (Emsley et al. 2010). The N-terminal residues of the bird adenovirus Rep that do not fit into the map were subsequently removed, resulting in a Rep monomer subunit with 6-270 amino acids. To complete the hexamer model, 5 monomeric subunits were generated through the same process.

### Identification of human-specific structural clusters

Human host annotations of 4,462 viruses were assigned using both NCBI virus (July 2022) and the Uniprot taxonomy (downloaded 24 July 2025, extracting viruses with host label 9606) (The UniProt Consortium 2017), where viruses linked to human hosts by at least 1 database were deemed human infecting. The prevalence of each structural cluster in human and non-human infecting viruses was calculated for each viral family. We defined a cluster human enrichment score per family as the prevalence frequency of the cluster in human infecting groups—the cluster prevalence in non-human groups (thus, the score ranged from −1 to 1). We filtered for structural clusters that were present in 2 or more viral families with a non-zero enrichment score. Six structural clusters were enriched in human-infecting viruses, and 5 structural clusters were enriched in non-human infecting viruses (Fig. 4d). We performed a Fisher’s exact test to examine if tropism was associated with the presence of a structural cluster and applied FDR correction to obtain q-values. Additionally, to perform a follow-up analysis on whether viruses were enriched in primates in general, we repeated the above analysis by adding primate host labels from NCBI virus (taxid:9443).

### Within-family human-tropism classification via KNN models

We used a K-nearest neighbors’ model to predict virus host tropism. K was originally set to 5, the median number of human-infecting viruses in a family (Figure S9h). We also tested *k* = 3 and *k* = 7, but these either had a lower AUROC or excluded many viral families (Figures S9i,j). The Jaccard index was used as the distance metric. For viral families with ≥ 5 human-infecting viruses, KNN classifiers were trained using viral structural encodings to distinguish human-infecting from nonhuman-infecting viruses, employing a leave-one-out process as described previously. AUROC was computed for each family. Seven family-specific predictors achieved an AUROC above 0.7 for predicting human tropism, and members of the “human” class were classified correctly at least once.

### Extracting structural features predictive of human-infecting viruses

To identify critical features for human classification, we estimated the abundance of structural features within 4 classes, defined by whether the virus was correctly classified and whether the virus was human tropic. The abundance was calculated as the count of the structural cluster within each class, normalized by the number of members. Structural clusters were manually chosen as human-specific if they were highly abundant in correctly classified human-infecting viruses, and often human predictive features were not present at all in non-human infecting viruses (Figures S6c,d, e).

### Structural alignment of E4 proteins

Structures predictive of human-tropism were modeled to examine features present in human vs non-human infecting viruses. Two E4 structural cluster representatives were chosen, each from human-infecting *Gammapapillomaviruses* and non-human infecting viruses. The E4 clusters chosen as representatives were the 2 most abundant in human and non-human infecting viruses. PDB files of the structure representative of each cluster were downloaded from the Colab notebook: https://colab.research.google.com/github/jnoms/vpSAT/blob/main/bin/colab/ExploreStructures.ipynb#scrollTo=OFQ-WsEEvHTr. Structural alignments were conducted using the PyMOL Molecular Graphics System, Version 3.0, Schrödinger, LLC. All structures were aligned relative to the folded structure of YP_004934016, the representative of cluster 699. The last 50 residues of each structure were aligned to regions 115-170 of the representative with the super command.

### Predicting intrinsic disorder in *Papillomaviridae* E4 proteins from amino acid sequence

IUPred2A was downloaded from https://iupred2a.elte.hu/download_new on January 28, 2026, and used to predict intrinsic disorder on all 94 E4 amino acid sequences using the long parameter. flDPnn (release version December 2021) was installed within an Apptainer container v1.2.2-1.el8. All E4 amino acid sequences were run through the intrinsic disorder prediction only function.

### Putative E4 annotation

Five *Gammapapillomaviruses,* which were missing E4 annotations, were explored for putative E4. Since E4 is found as an internal open reading frame of E2 (Doorbar 2013), E2's coding sequence was queried for internal open reading frames using NCBI ORFfinder. ORFs above 200 bp were considered as potentially coding for E4. The putative E4 ORFs were queried using BLASTP with BLOSUM45 to look for distant sequence homology (Altschul et al. 1990). Multiple sequence alignment was performed by MAFFT v7.490 (Katoh et al. 2002). Alignments were manually inspected for conserved domains using Geneious Prime 2025.2.1. Three of the 4 putative E4s contained domains (proline-rich regions, negatively charged domains, and positively charged domains) characteristic of E4 proteins. The 3 E4s with the expected domain architecture were folded with Alphafold3 (Abramson et al. 2024). The identified putative ORFs and their genomic coordinates are provided in Supplementary Dataset S3.

### Statistical analysis and visualization

Boxplots display the median (center line), the 25th and 75th percentiles (box edges), and whiskers extending to the 1.5*IQR range. Phi coefficients were calculated to measure the mutual exclusivity of structural clusters in a virus, and Fisher's exact test was used to estimate significance. Correlation coefficients and *P*-values were estimated using Spearman's rank correlation. AUROC was calculated using the pROC package (v1.19.0.1) in R. Differences in performance with different hyperparameters were modeled with a linear mixed effects model built with lme4 (v1.1.37), and significance of model variables was determined by the likelihood ratio test. In applicable cases, the false discovery rate was applied to correct for multiple hypothesis testing.

## Supplementary Material

msag110_Supplementary_Data

## Data Availability

All code is available on GitHub: https://github.com/AuslanderLab/Evol-structural-virus. All intermediate files and code are available on Zenodo: https://zenodo.org/records/17610725.
