# Supplementary material for: Uncovering viral protein acquisition events and human-specific folds with pairwise comparisons of predicted protein structures
Source: Mol Biol Evol. 2026 Apr 27;43(5):msag110. doi: 10.1093/molbev/msag110 (PMC13172254; doi:10.1093/molbev/msag110)
Supplement: msag110_Supplementary_Data [file msag110_supplementary_data.zip › supplementary_file_evol_virome.pdf]

Supporting Information Text for Uncovering viral protein acquisition events and human-specific folds with pairwise comparisons of predicted protein structures

Supplementary Methods

Building a tree to visualize protein fold sharing networks

To visualize the relationships captured in viral structural encodings, a distance matrix was constructed with the metric 1- $J_{J,K}$, where $J_{J,K}$ is defined as $J_{J,K} = \frac{x_{J} \cap x_{K}}{x_{J} \cup x_{K}}$ and x_j and x_k are viral structural encodings of two distinct virus species. The dendrogram was computed using hierarchical clustering with complete linkage. Visualization of the tree was performed with ggtree (Xu et al. 2022) (v3.12.0) and ggtreeExtras (Xu et al. 2021) (version 1.14.0). When analyzing viruses that shared protein folds with Redondoviruses (Supplementary Figure 4B), 713 viruses containing the structural clusters 4, 3032, and 11523, were selected.

Supplemental Figures

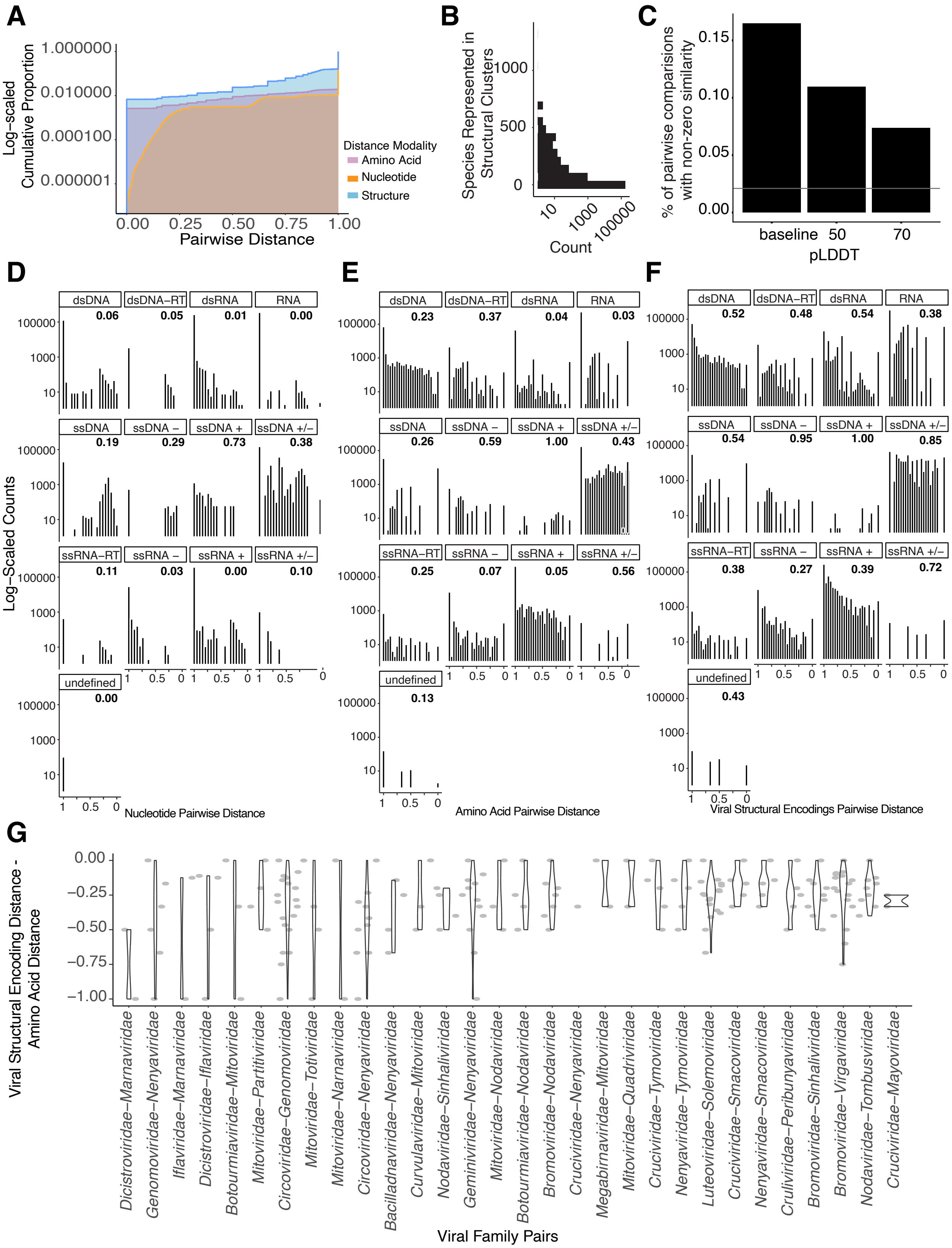

Fig S1. A Log-scaled empirical cumulative distribution of pairwise distances between viral proteins across eukaryotic viruses with RefSeq proteins from the Nomburg et al. dataset, computed using nucleotide (orange), amino acid (pink), and viral structural encodings (blue) . **B** Count histogram presenting the number of species represented in each structural cluster (bin size=20) in the Nomburg et al. dataset. **C** Proportion of pairwise distances that are smaller than one (y-axis) across virus pairs from the Nomburg et al. dataset, including high-confidence structures in the viral structural encodings, with thresholds for high confidence structures (x-axis), 0 (baseline, all structures), 50, and 70. (y-axis). Grey line indicates the 2.4% of pairs comparable with amino acid similarity. **D,E,F** Log-scaled count distribution of pairwise distances between viruses within each molecule representation (box). Pairwise Distances are computed from nucleotide sequences (D), amino acid sequences (E), and viral structural encodings (x-axes). **G** Top 30 inter-familial comparisons with the greatest average increase of structural similarity are shown.

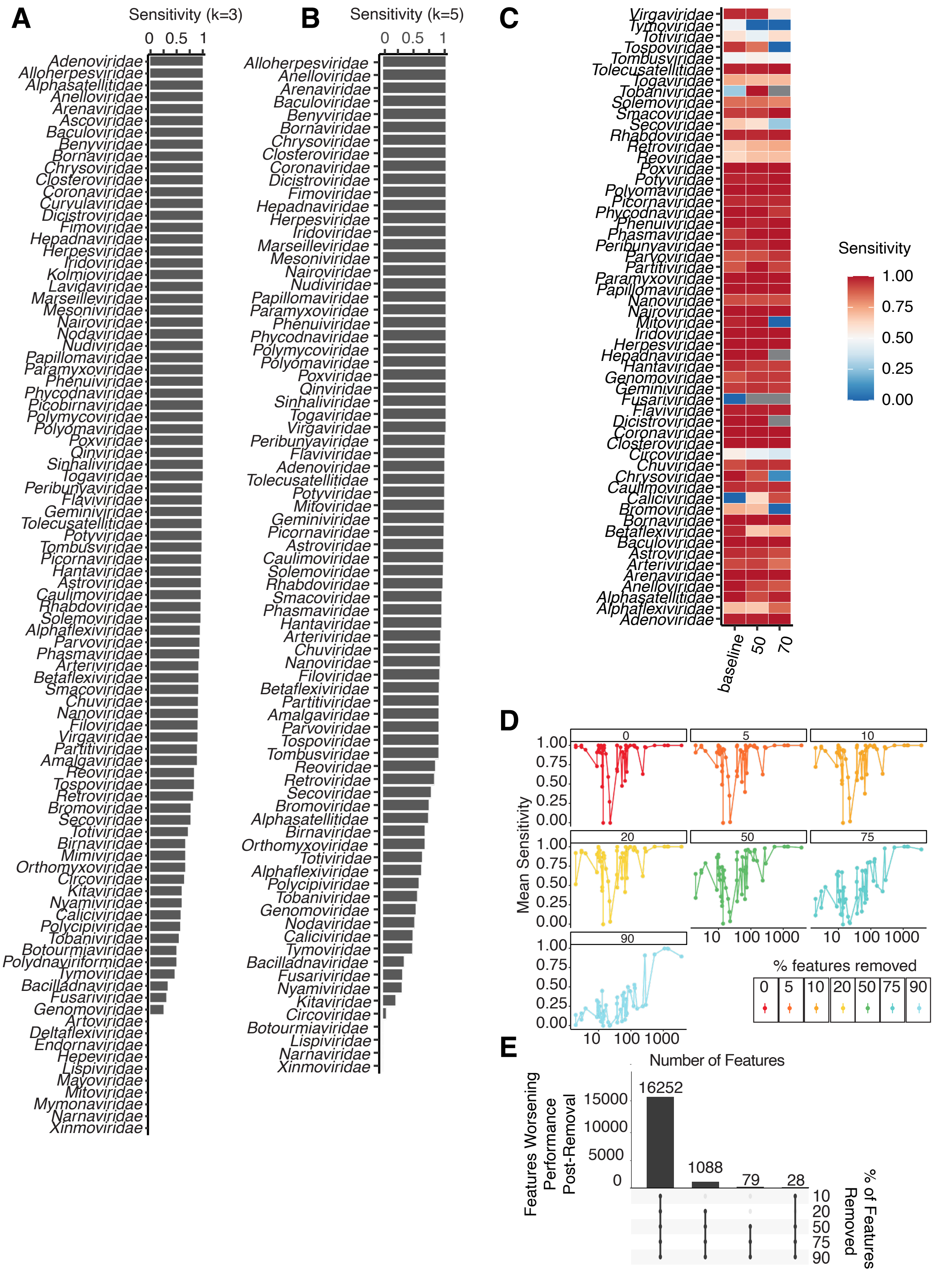

A
Sensitivity (k=3)
0 0.5 1
Adenoviridae
Alloherpesviridae
Alphasatellitidae
Anelloviridae
Arenaviridae
Ascoviridae
Baculoviridae
Benyviridae
Bornaviridae
Chrysoviridae
Closteroviridae
Coronaviridae
Curvulaviridae
Dicistroviridae
Fimoviridae
Hepadnaviridae
Herpesviridae
Iridoviridae
Kolmioviridae
Lavidaviridae
Marseilleviridae
Mesoniviridae
Nairoviridae
Nodaviridae
Nudiviridae
Papillomaviridae
Paramyxoviridae
Phenuiviridae
Phycodnaviridae
Picobirnaviridae
Polymycoviridae
Polyomaviridae
Poxviridae
Qinviridae
Sinhaliviridae
Togaviridae
Peribunyaviridae
Flaviviridae
Geminiviridae
Tolecusatellitidae
Potyviridae
Tombusviridae
Picornaviridae
Hantaviridae
Astroviridae
Caulimoviridae
Rhabdoviridae
Solemoviridae
Alphaflexiviridae
Parvoviridae
Phasmaviridae
Arteriviridae
Betaflexiviridae
Smacoviridae
Chuviridae
Nanoviridae
Filoviridae
Virgaviridae
Partitiviridae
Amalgaviridae
Reoviridae
Tospoviridae
Retroviridae
Bromoviridae
Secoviridae
Totiviridae
Birnaviridae
Mimiviridae
Orthomyxoviridae
Circoviridae
Kitaviridae
Nyamiviridae
Caliciviridae
Polycipiviridae
Tobaniviridae
Botourmiaviridae
Polydnaviriformidae
Tymoviridae
Bacilladnaviridae
Fusariviridae
Genomoviridae
Artoviridae
Deltaflexiviridae
Endornaviridae
Hepeviridae
Lispiviridae
Mayoviridae
Mitoviridae
Mymonaviridae
Narnaviridae
Xinmoviridae
B
Sensitivity (k=5)
0 0.5 1
Alloherpesviridae
Anelloviridae
Arenaviridae
Baculoviridae
Benyviridae
Bornaviridae
Chrysoviridae
Closteroviridae
Coronaviridae
Dicistroviridae
Fimoviridae
Hepadnaviridae
Herpesviridae
Iridoviridae
Marseilleviridae
Mesoniviridae
Nairoviridae
Nudiviridae
Papillomaviridae
Paramyxoviridae
Phenuiviridae
Phycodnaviridae
Polymycoviridae
Polyomaviridae
Poxviridae
Qinviridae
Sinhaliviridae
Togaviridae
Virgaviridae
Peribunyaviridae
Flaviviridae
Adenoviridae
Tolecusatellitidae
Potyviridae
Mitoviridae
Geminiviridae
Picornaviridae
Astroviridae
Caulimoviridae
Solemoviridae
Rhabdoviridae
Smacoviridae
Phasmaviridae
Hantaviridae
Arteriviridae
Chuviridae
Nanoviridae
Filoviridae
Betaflexiviridae
Partitiviridae
Amalgaviridae
Parvoviridae
Tospoviridae
Tombusviridae
Reoviridae
Retroviridae
Secoviridae
Bromoviridae
Alphasatellitidae
Birnaviridae
Orthomyxoviridae
Totiviridae
Alphaflexiviridae
Polycipiviridae
Tobaniviridae
Genomoviridae
Nodaviridae
Caliciviridae
Tymoviridae
Bacilladnaviridae
Fusariviridae
Nyamiviridae
Kitaviridae
Circoviridae
Botourmiaviridae
Lispiviridae
Narnaviridae
Xinmoviridae
C
Virgaviridae
Tymoviridae
Totiviridae
Tospoviridae
Tombusviridae
Tolecusatellitidae
Togaviridae
Tobaniviridae
Solemoviridae
Smacoviridae
Secoviridae
Rhabdoviridae
Retroviridae
Reoviridae
Poxviridae
Potyviridae
Polyomaviridae
Picornaviridae
Phycodnaviridae
Phenuiviridae
Phasmaviridae
Peribunyaviridae
Parvoviridae
Partitiviridae
Paramyxoviridae
Papillomaviridae
Nanoviridae
Nairoviridae
Mitoviridae
Iridoviridae
Herpesviridae
Hepadnaviridae
Hantaviridae
Genomoviridae
Geminiviridae
Fusariviridae
Flaviviridae
Dicistroviridae
Coronaviridae
Closteroviridae
Circoviridae
Chuviridae
Chrysoviridae
Caulimoviridae
Caliciviridae
Bromoviridae
Bornaviridae
Betaflexiviridae
Baculoviridae
Astroviridae
Arteriviridae
Arenaviridae
Anelloviridae
Alphasatellitidae
Alphaflexiviridae
Adenoviridae
baseline
50
70
Sensitivity
1.00
0.75
0.50
0.25
0.00
D
Mean Sensitivity
0
5
10
20
50
75
90
1.00
0.75
0.50
0.25
0.00
10 100 1000
% features removed
0 5 10 20 50 75 90
E
Number of Features
Features Worsening Performance Post-Removal
15000
10000
5000
0
16252
1088
79
28
% of Features Removed
10
20
50
75
90

Fig. S2. A,B Sensitivity of viral family classification using virus structural encodings, with hyperparameters k=3 (A) and k=5 (B). **C** Average viral family classification sensitivity for different families (y-axis)across different pLDDT confidence thresholds (x-axis) used as criteria for inclusion as input features (if confidence is greater than pLDDT threshold). **D** Comparison of the mean number of features per family (x-axis) and the mean sensitivity across all iterations of bootstrapped feature removal (y-axis). Each point is a viral family. Color indicates the percentage of features removed. **E** Upset plot of the number of features (y-axis, left) associated with worsening performance in multiple removal batches (y-axis, right).

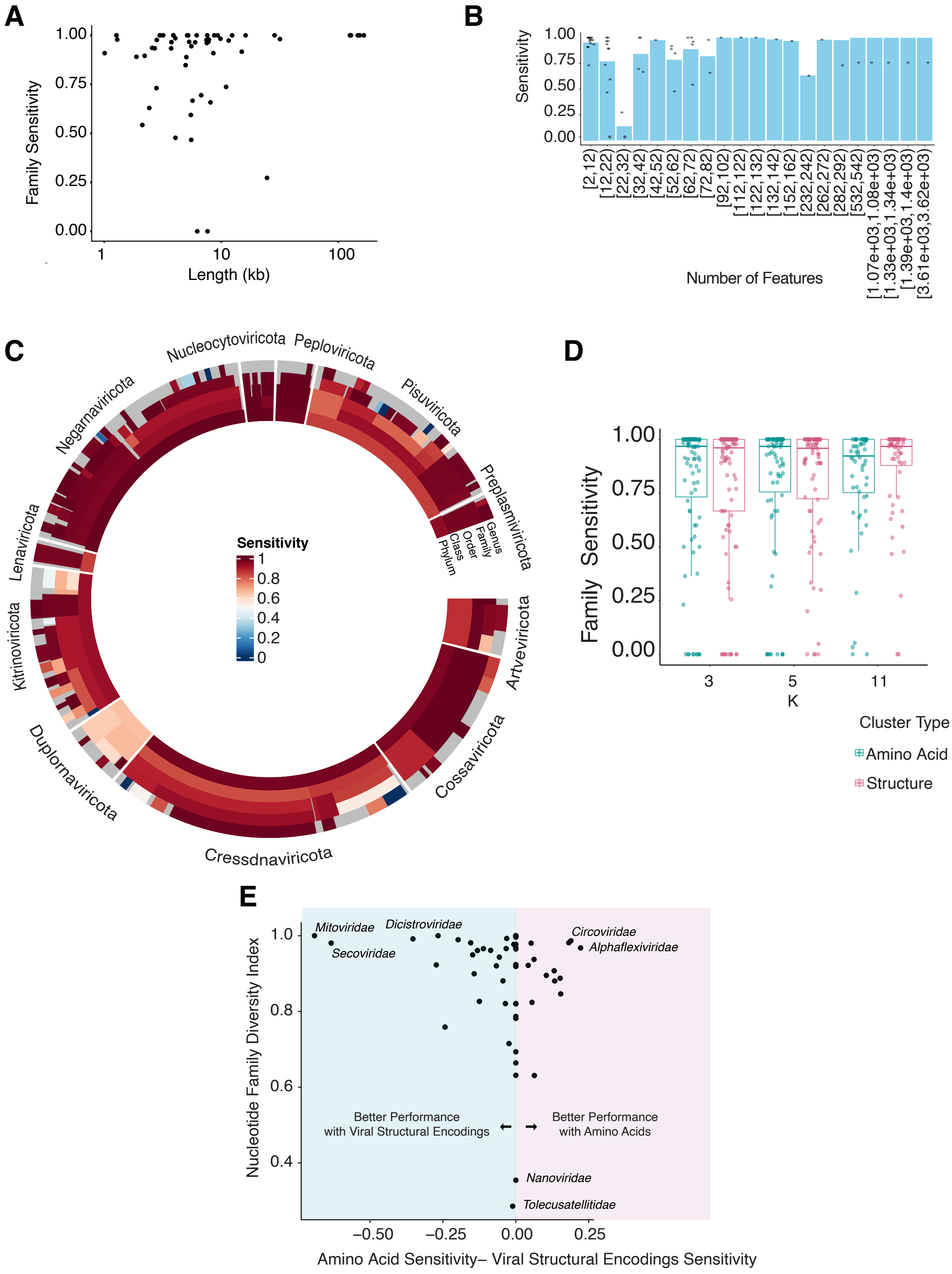
A
Family Sensitivity
1.00
0.75
0.50
0.25
0.00
1
10
100
Length (kb)
B
Sensitivity
1.00
0.75
0.50
0.25
0.00
[2,12)
[12,22)
[22,32)
[32,42)
[42,52)
[52,62)
[62,72)
[72,82)
[92,102)
[112,122)
[122,132)
[132,142)
[152,162)
[232,242)
[262,272)
[282,292)
[532,542)
[1.07e+03,1.08e+03)
[1.33e+03,1.34e+03)
[1.39e+03,1.4e+03)
[3.61e+03,3.62e+03)
Number of Features
C
Nucleocytoviricota
Peploviricota
Pisuviricota
Preplasmiviricota
Negarnaviricota
Lenarviricota
Kitrinoviricota
Duplornaviricota
Cressdnaviricota
Cossaviricota
Artverviricota
Genus
Family
Order
Class
Phylum
Sensitivity
1
0.8
0.6
0.4
0.2
0
D
Family Sensitivity
1.00
0.75
0.50
0.25
0.00
3
5
11
K
Cluster Type
Amino Acid
Structure
E
Nucleotide Family Diversity Index
1.0
0.8
0.6
0.4
Mitoviridae
Dicistroviridae
Secoviridae
Circoviridae
Alphaflexiviridae
Better Performance with Viral Structural Encodings
Better Performance with Amino Acids
Nanoviridae
Tolecusatellitidae
−0.50
−0.25
0.00
0.25
Amino Acid Sensitivity– Viral Structural Encodings Sensitivity

Fig. S3. A Scatterplot of the viral family classification sensitivity (y-axis) vs the average genome size in kB (x-axis). Each dot represents a family. **B** Barplot showing for the number of features in a family (x-axis, binned in intervals of 10) the average family sensitivity (y-axis). Each point represents an individual family. **C** Sensitivity of virus classification into different taxonomic levels as in Figure 2D, where circle sections are grouped at the phylum level, but also including species with insufficient members to classify at a specific taxonomic level (in grey). **D** Boxplot of family sensitivity using amino acids (green) or structural encodings (pink). The family sensitivity across different hyperparameters (x-axis) used for classification. Differences were not statistically significant. **E** The difference in family classification sensitivity when using amino acid features vs. structural features (x-axis) compared to the average within-family nucleotide distance (y-axis). Each point represents a viral family.

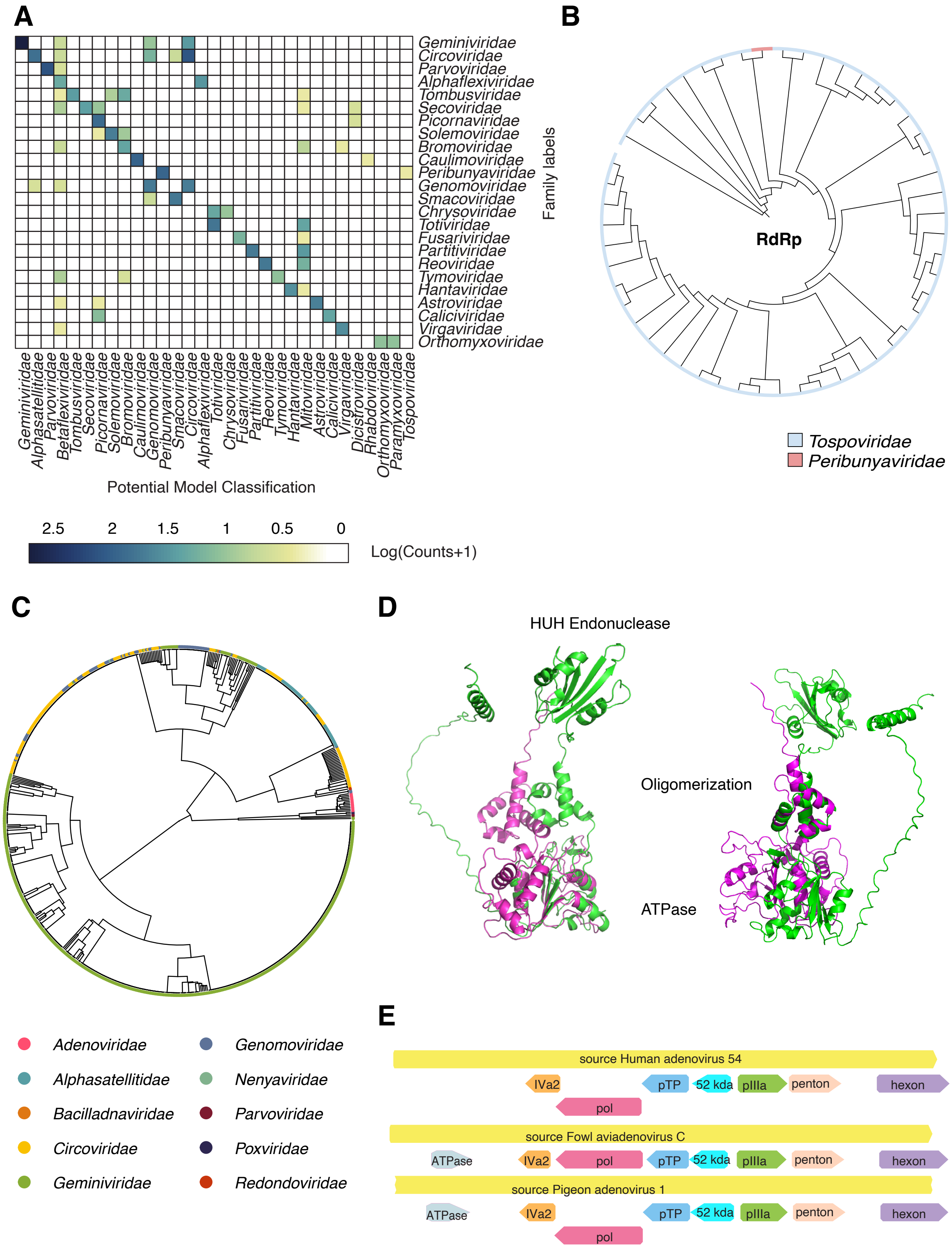

Fig. S4. A Heatmap of viral families (y-axis) and the potential misclassified into (x-axis). The coloring in the

heatmap represents the number of viruses in the family (y-axis) which could be classified into each other family (x-axis) based on the 11 nearest neighbors. Ties were included in the counts, which are log-transformed. **B** RdRp tree built using the top 50 BLASTp hits (by E-value) when using YP_009126736.1, the L protein of the Mulberry vein banding virus, sequence as query. **C** Phylogenetic tree representation built using 420 sequences of viruses that share protein folds with Redondovirus. Each point is colored by the viral family label. **D** Structural alignment of a canonical ssDNA virus Rep (YP_009237536, pLDDT=75.59) to ORF2/Rep protein in Pidgeon adenovirus (YP_009047088, pLDDT= 88.68). Presented are the alignment of the ATPase domain (top) and alignment of the oligomerization domain (bottom). **E** Mock genome plot of human adenovirus (top) and avian adenoviruses (bottom two). Newly discovered Rep shown as the grey protein. Denoted in orange are Iva2 proteins, the ATPase present in all members of *Adenoviridae*.

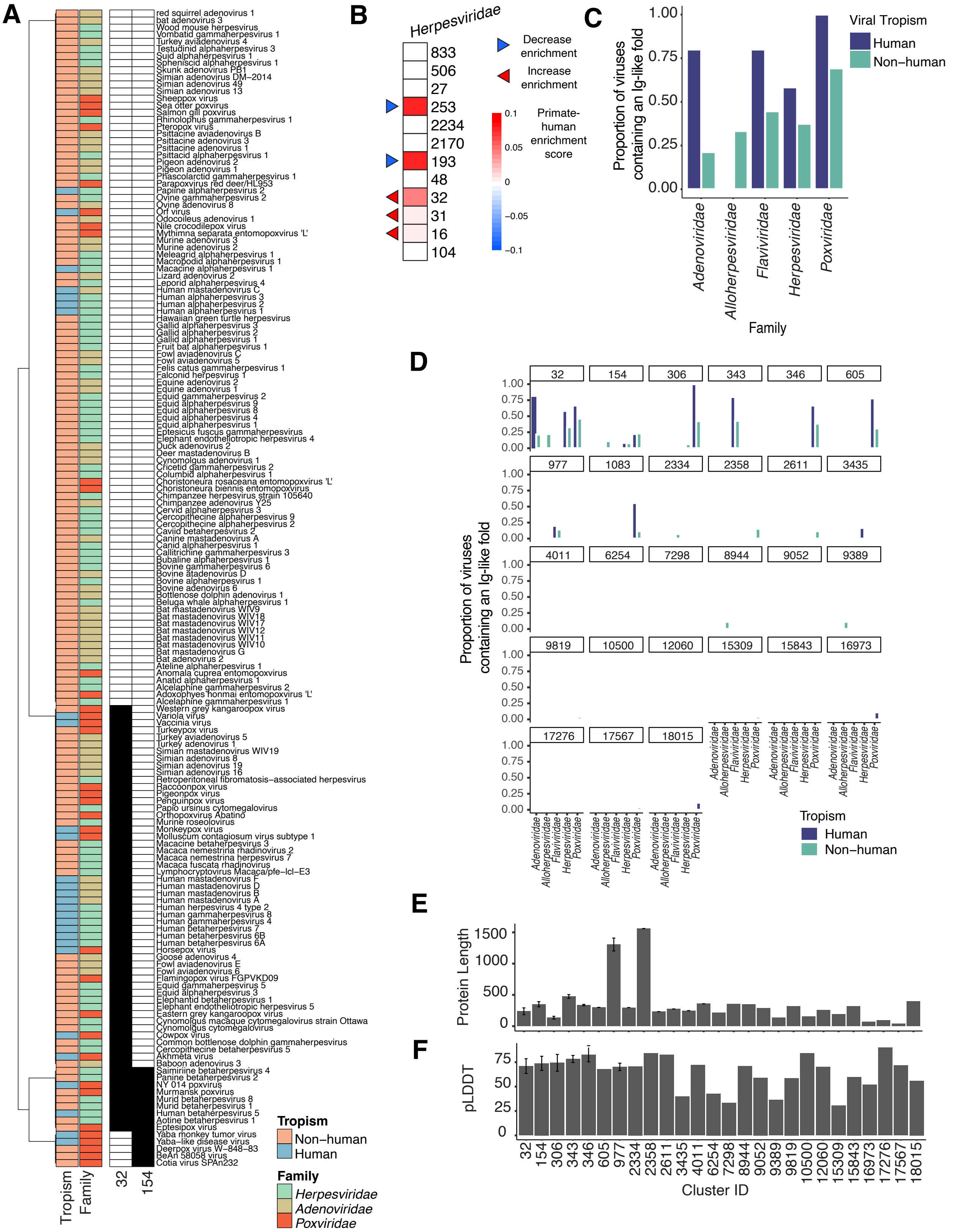
A
red squirrel adenovirus 1
bat adenovirus 3
Wood mouse herpesvirus
Vombatid gammaherpesvirus 1
Turkey aviadenovirus 4
Testudinid alphaherpesvirus 3
Suid alphaherpesvirus 1
Spheniscid alphaherpesvirus 1
Skunk adenovirus PB1
Simian adenovirus DM-2014
Simian adenovirus 49
Simian adenovirus 13
Sheeppox virus
Sea otter poxvirus
Salmon gill poxvirus
Rhinolophus gammaherpesvirus 1
Pteropox virus
Psittacine aviadenovirus B
Psittacine adenovirus 3
Psittacine adenovirus 1
Psittacid alphaherpesvirus 1
Pigeon adenovirus 2
Pigeon adenovirus 1
Phascolarctid gammaherpesvirus 1
Parapoxvirus red deer/HL953
Papiine alphaherpesvirus 2
Ovine gammaherpesvirus 2
Ovine adenovirus 8
Orf virus
Odocoileus adenovirus 1
Nile crocodilepox virus
Mythimna separata entomopoxvirus 'L'
Murine adenovirus 3
Murine adenovirus 2
Meleagrid alphaherpesvirus 1
Macropodid alphaherpesvirus 1
Macacine alphaherpesvirus 1
Lizard adenovirus 2
Leporid alphaherpesvirus 4
Human mastadenovirus C
Human alphaherpesvirus 3
Human alphaherpesvirus 2
Human alphaherpesvirus 1
Hawaiian green turtle herpesvirus
Gallid alphaherpesvirus 3
Gallid alphaherpesvirus 2
Gallid alphaherpesvirus 1
Fruit bat alphaherpesvirus 1
Fowl aviadenovirus C
Fowl aviadenovirus 5
Felis catus gammaherpesvirus 1
Falconid herpesvirus 1
Equine adenovirus 2
Equine adenovirus 1
Equid gammaherpesvirus 2
Equid alphaherpesvirus 9
Equid alphaherpesvirus 8
Equid alphaherpesvirus 4
Equid alphaherpesvirus 1
Eptesicus fuscus gammaherpesvirus
Elephant endotheliotropic herpesvirus 4
Duck adenovirus 2
Deer mastadenovirus B
Cynomolgus adenovirus 1
Cricetid gammaherpesvirus 2
Columbid alphaherpesvirus 1
Choristoneura rosaceana entomopoxvirus 'L'
Choristoneura biennis entomopoxvirus
Chimpanzee herpesvirus strain 105640
Chimpanzee adenovirus Y25
Cervid alphaherpesvirus 3
Cercopithecine alphaherpesvirus 9
Cercopithecine alphaherpesvirus 2
Caviid betaherpesvirus 2
Canine mastadenovirus A
Canid alphaherpesvirus 1
Callitrichine gammaherpesvirus 3
Bubaline alphaherpesvirus 1
Bovine gammaherpesvirus 6
Bovine atadenovirus D
Bovine alphaherpesvirus 1
Bovine adenovirus 6
Bottlenose dolphin adenovirus 1
Beluga whale alphaherpesvirus 1
Bat mastadenovirus WIV9
Bat mastadenovirus WIV18
Bat mastadenovirus WIV17
Bat mastadenovirus WIV12
Bat mastadenovirus WIV11
Bat mastadenovirus WIV10
Bat mastadenovirus G
Bat adenovirus 2
Ateline alphaherpesvirus 1
Anomala cuprea entomopoxvirus
Anatid alphaherpesvirus 1
Alcelaphine gammaherpesvirus 2
Adoxophyes honmai entomopoxvirus 'L'
Alcelaphine gammaherpesvirus 1
Western grey kangaroopox virus
Variola virus
Vaccinia virus
Turkeypox virus
Turkey aviadenovirus 5
Turkey adenovirus 1
Simian mastadenovirus WIV19
Simian adenovirus 8
Simian adenovirus 19
Simian adenovirus 16
Retroperitoneal fibromatosis-associated herpesvirus
Raccoonpox virus
Pigeonpox virus
Penguinpox virus
Papio ursinus cytomegalovirus
Orthopoxvirus Abatino
Murine roseolovirus
Monkeypox virus
Molluscum contagiosum virus subtype 1
Macacine betaherpesvirus 3
Macaca nemestrina rhadinovirus 2
Macaca nemestrina herpesvirus 7
Macaca fuscata rhadinovirus
Lymphocryptovirus Macaca/pfe-lcl-E3
Human mastadenovirus F
Human mastadenovirus D
Human mastadenovirus B
Human mastadenovirus A
Human herpesvirus 4 type 2
Human gammaherpesvirus 8
Human gammaherpesvirus 4
Human betaherpesvirus 7
Human betaherpesvirus 6B
Human betaherpesvirus 6A
Horsepox virus
Goose adenovirus 4
Fowl aviadenovirus E
Fowl aviadenovirus 6
Flamingopox virus FGPVKD09
Equid gammaherpesvirus 5
Equid alphaherpesvirus 3
Elephantid betaherpesvirus 1
Elephant endotheliotropic herpesvirus 5
Eastern grey kangaroopox virus
Cynomolgus macaque cytomegalovirus strain Ottawa
Cynomolgus cytomegalovirus
Cowpox virus
Common bottlenose dolphin gammaherpesvirus
Cercopithecine betaherpesvirus 5
Akhmeta virus
Baboon adenovirus 3
Saimiriine betaherpesvirus 4
Panine betaherpesvirus 2
NY 014 poxvirus
Murmansk poxvirus
Murid betaherpesvirus 8
Murid betaherpesvirus 1
Human betaherpesvirus 5
Aotine betaherpesvirus 1
Eptesipox virus
Yaba monkey tumor virus
Yaba-like disease virus
Deerpox virus W-848-83
BeAn 58058 virus
Cotia virus SPAn232
Tropism
Family
32
154
Tropism
Non-human
Human
Family
Herpesviridae
Adenoviridae
Poxviridae
B
Herpesviridae
833
506
27
253
2234
2170
193
48
32
31
16
104
Decrease enrichment
Increase enrichment
Primate-human enrichment score
0.1
0.05
0
−0.05
−0.1
C
Proportion of viruses containing an Ig-like fold
1.00
0.75
0.50
0.25
0.00
Viral Tropism
Human
Non-human
Adenoviridae
Alloherpesviridae
Flaviviridae
Herpesviridae
Poxviridae
Family
D
Proportion of viruses containing an Ig-like fold
32
154
306
343
346
605
977
1083
2334
2358
2611
3435
4011
6254
7298
8944
9052
9389
9819
10500
12060
15309
15843
16973
17276
17567
18015
1.00
0.75
0.50
0.25
0.00
Adenoviridae
Alloherpesviridae
Flaviviridae
Herpesviridae
Poxviridae
Tropism
Human
Non-human
E
Protein Length
1500
1000
500
0
F
pLDDT
75
50
25
0
32
154
306
343
346
605
977
2334
2358
2611
3435
4011
6254
7298
8944
9052
9389
9819
10500
12060
15309
15843
16973
17276
17567
18015
Cluster ID

60

Fig. S5 A Heatmap showing presence vs absence of clusters 32 and 154 across Herpesviridae, Adenoviridae, and poxviridae members in the dataset. Colored legends indicate viral tropism (left) and viral family (right). **B** Heatmap showing changes in enrichment score between human-infecting and primate-infecting viruses (colormap represents the difference) for different clusters (y-axis) within Herpesviridae. Arrows indicate the direction of the effect relative to human enrichment scores. **C** The proportion (y-axis) of viruses having one or more proteins belonging to a structural cluster with an immunoglobulin domain annotated member. The proportion is calculated within families (x-axis). **D** The proportion of viruses (y-axes) within families and tropism (x-axis) containing each specific structural cluster (facet label). **E ,F** Bar plots with showing the average and ±1 standard deviation of protein lengths (E) and pLDDT (F) for each structural cluster with an immunoglobulin domain annotated member

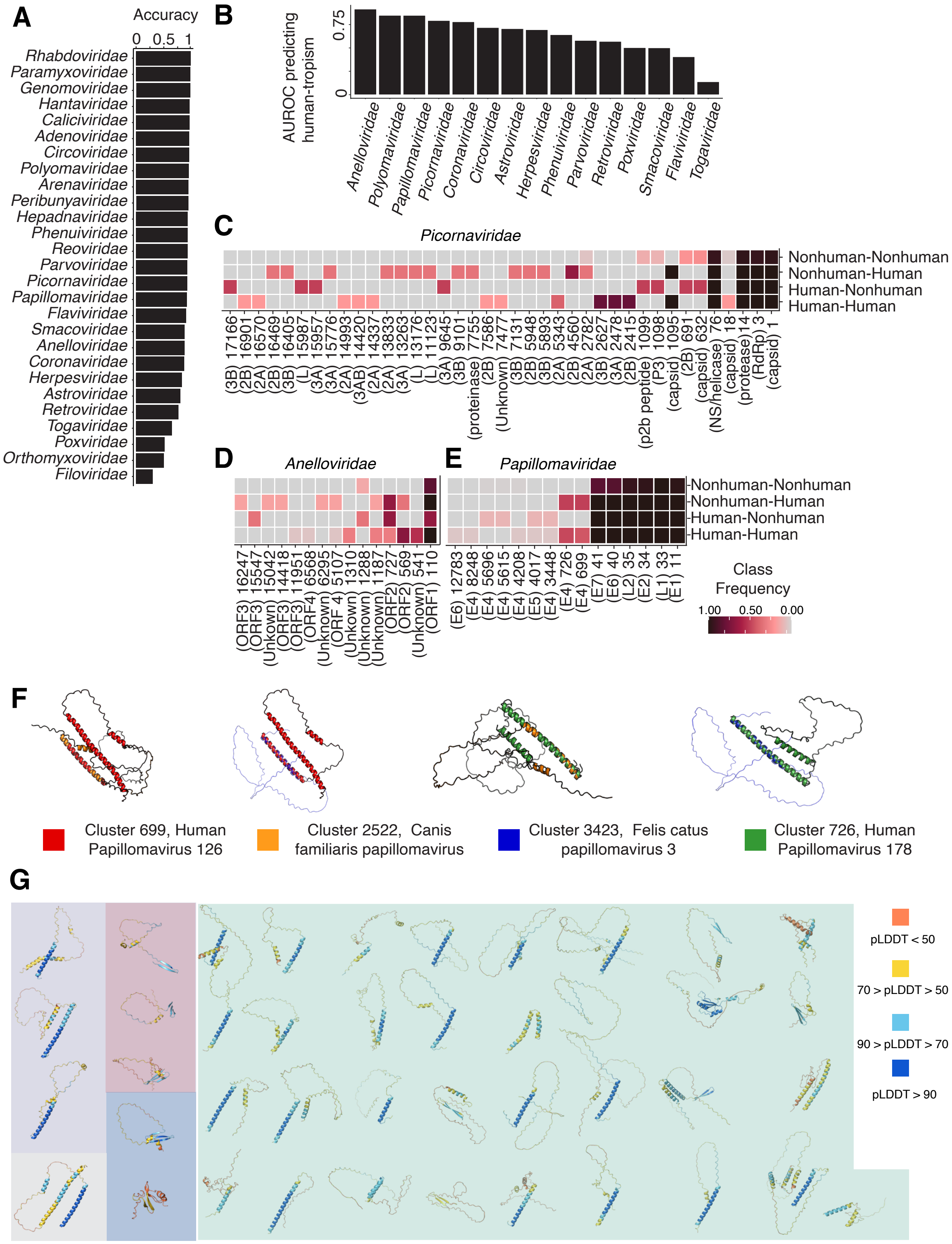
A
Accuracy
0 0.5 1
Rhabdoviridae
Paramyxoviridae
Genomoviridae
Hantaviridae
Caliciviridae
Adenoviridae
Circoviridae
Polyomaviridae
Arenaviridae
Peribunyaviridae
Hepadnaviridae
Phenuiviridae
Reoviridae
Parvoviridae
Picornaviridae
Papillomaviridae
Flaviviridae
Smacoviridae
Anelloviridae
Coronaviridae
Herpesviridae
Astroviridae
Retroviridae
Togaviridae
Poxviridae
Orthomyxoviridae
Filoviridae
B
AUROC predicting human-tropism
0
0.75
Anelloviridae
Polyomaviridae
Papillomaviridae
Picornaviridae
Coronaviridae
Circoviridae
Astroviridae
Herpesviridae
Phenuiviridae
Parvoviridae
Retroviridae
Poxviridae
Smacoviridae
Flaviviridae
Togaviridae
C
Picornaviridae
Nonhuman-Nonhuman
Nonhuman-Human
Human-Nonhuman
Human-Human
(3B) 17166
(2B) 16901
(2A) 16570
(2B) 16469
(3B) 16405
(L) 15987
(3A) 15957
(3A) 15776
(2A) 14993
(3AB) 14420
(2A) 14337
(2A) 13833
(3A) 13263
(L) 13176
(L) 11123
(3A) 9645
(3B) 9101
(proteinase) 7755
(2B) 7586
(Unknown) 7477
(3B) 7131
(2B) 5948
(3B) 5893
(2A) 5343
(2B) 4560
(2A) 2782
(3B) 2627
(3A) 2478
(2B) 2415
(p2b peptide) 1099
(P3) 1098
(capsid) 1095
(2B) 691
(capsid) 632
(NS/helicase) 76
(capsid) 18
(protease) 14
(RdRp) 3
(capsid) 1
D
Anelloviridae
(ORF3) 16247
(ORF3) 15547
(Unkown) 15042
(ORF3) 14418
(ORF3) 11951
(ORF4) 6568
(Unknown) 6295
(ORF 4) 5107
(Unkown) 1310
(Unknown) 1288
(Unknown) 1187
(ORF2) 727
(ORF2) 569
(Unknown) 541
(ORF1) 110
E
Papillomaviridae
Nonhuman-Nonhuman
Nonhuman-Human
Human-Nonhuman
Human-Human
(E6) 12783
(E4) 8248
(E4) 5696
(E4) 5615
(E4) 4208
(E5) 4017
(E4) 3448
(E4) 726
(E4) 699
(E7) 41
(E6) 40
(L2) 35
(E2) 34
(L1) 33
(E1) 11
Class Frequency
1.00 0.50 0.00
F
Cluster 699, Human Papillomavirus 126
Cluster 2522, Canis familiaris papillomavirus
Cluster 3423, Felis catus papillomavirus 3
Cluster 726, Human Papillomavirus 178
G
pLDDT < 50
70 > pLDDT > 50
90 > pLDDT > 70
pLDDT > 90

Fig. S6. A,B Accuracy (A) and AUROC (B) for prediction of human/non-human viral host using viruses within each viral family. **C,D**,E Heatmap visualizing the frequency of protein folds in four classes (y-axis); the classes are defined as the real label (left) - classifier label (right). The coloring is the abundance of the protein fold in each class which is calculated as the number of times a feature is present normalized by the number of members in each class. **F** The structural alignment of E4 in human and non-human papillomaviruses. **G** All E4 structural cluster representatives colored by pLDDT per residue. Grey box highlights the representative that all E4 structures were aligned to,YP_004934016, the cluster 699 *Gammapapillomavirus* representative. Purple boxes are used for E4 from *Gammapapillomaviruses*, blue boxes for E4 proteins from primate-infecting viruses, pink boxes for *Mupapillomavirus* and *Alphapapillomavirus E4* proteins, and green boxes for non–human-infecting *Papillomaviridae E4* proteins.

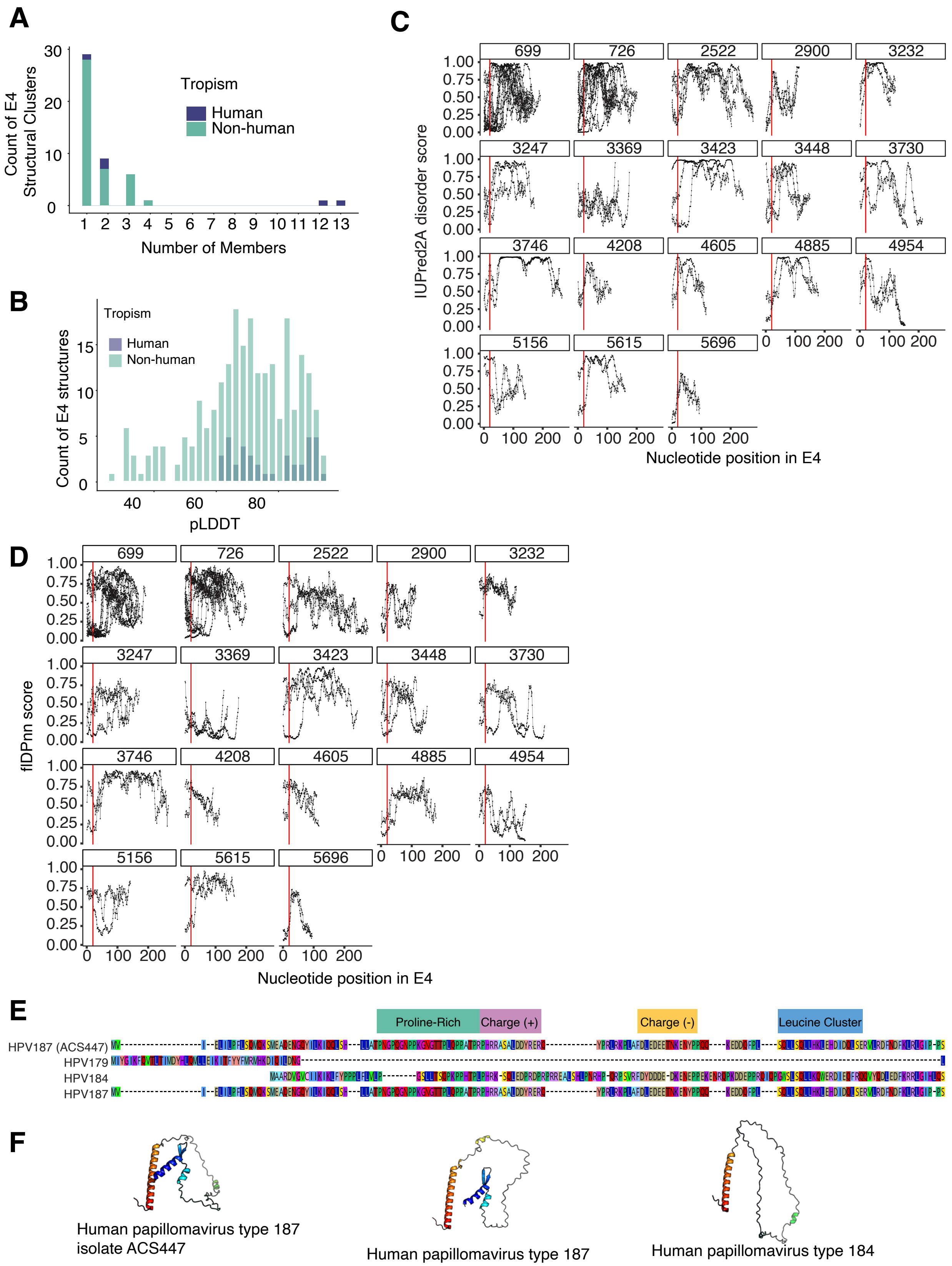
A
Count of E4 Structural Clusters
Tropism
Human
Non-human
Number of Members
B
Tropism
Human
Non-human
Count of E4 structures
pLDDT
C
IUPred2A disorder score
699
726
2522
2900
3232
3247
3369
3423
3448
3730
3746
4208
4605
4885
4954
5156
5615
5696
Nucleotide position in E4
D
fIDPnn score
699
726
2522
2900
3232
3247
3369
3423
3448
3730
3746
4208
4605
4885
4954
5156
5615
5696
Nucleotide position in E4
E
Proline-Rich
Charge (+)
Charge (-)
Leucine Cluster
HPV187 (ACS447)
HPV179
HPV184
HPV187
F
Human papillomavirus type 187 isolate ACS447
Human papillomavirus type 187
Human papillomavirus type 184

83

Fig. S7. **A** The counts (y-axis) of the number of members (x-axis) in each E4 structural cluster. Each cluster is colored by the tropism of majority of the viral members. **B** Histogram of pLDDT of every E4 protein structure (30 bins).**C, D,** **I**ntrinsic disorder scores (y-axes) predicted by flDPnn (G) and IUPred2A (H) for each position (x-axes) in the E4 sequences. Each facet represents a structural cluster, and each line represents a cluster member. Scores range from 0 to 1, where values closer to 1 indicate likely intrinsic disorder, and a score of 0 indicate a stable conformation. **E** Multiple sequence alignment of new E4 proteins which were annotated in RefSeq strains of *Gammapapillomavirus*. Domains commonly found in other E4 proteins are denoted on top. **F** The folded structures of the new E4 proteins of RefSeq strains of *Gammapapillomavirus*. The N terminal region is colored in blue shades, and oranges/reds indicate C terminal region.

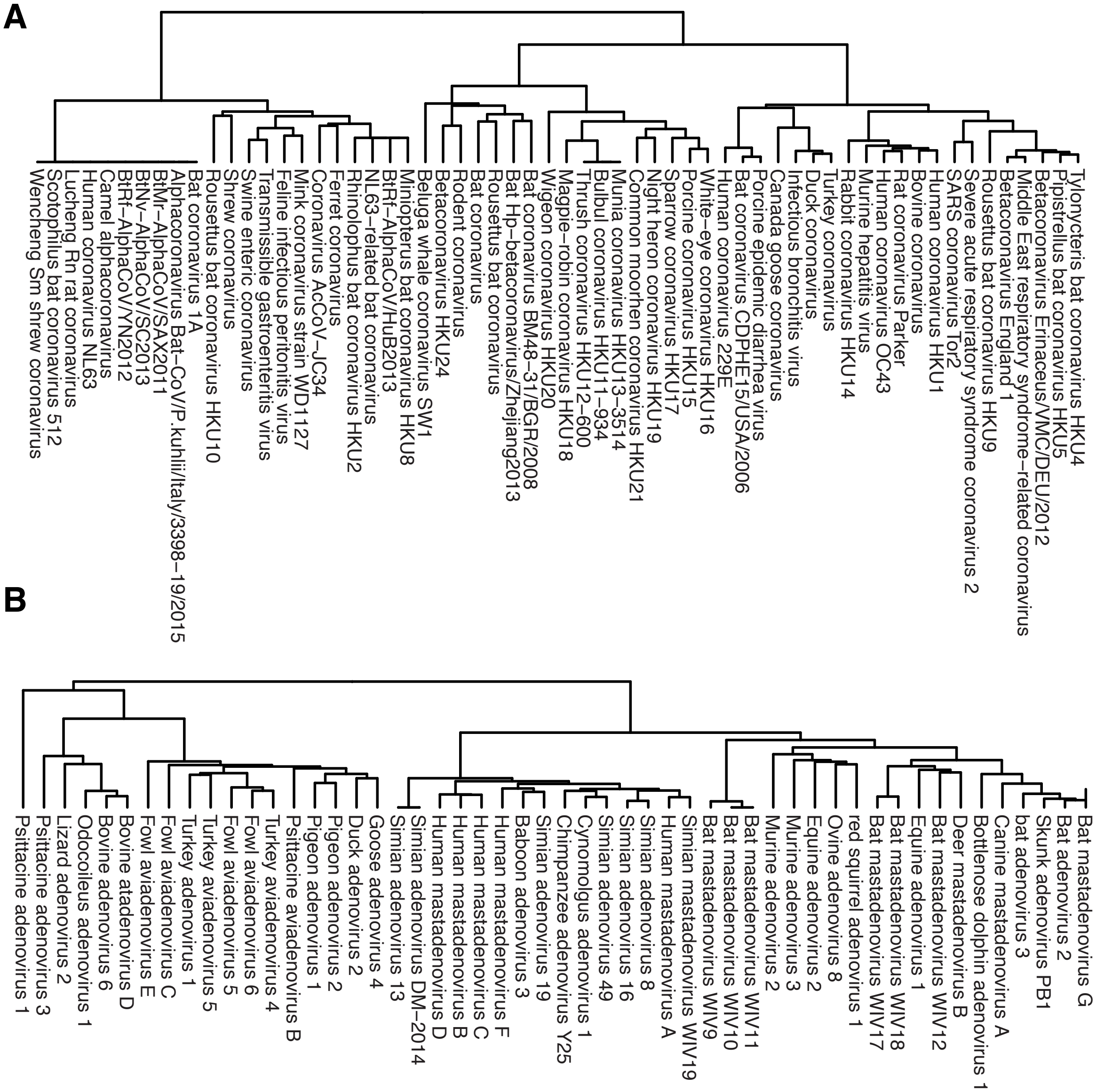
A
Wencheng Sm shrew coronavirus
Scotophilus bat coronavirus 512
Lucheng Rn rat coronavirus
Human coronavirus NL63
Camel alphacoronavirus
BtRf−AlphaCoV/YN2012
BtNv−AlphaCoV/SC2013
BtMr−AlphaCoV/SAX2011
Alphacoronavirus Bat−CoV/P.kuhlii/Italy/3398−19/2015
Bat coronavirus 1A
Rousettus bat coronavirus HKU10
Shrew coronavirus
Swine enteric coronavirus
Transmissible gastroenteritis virus
Feline infectious peritonitis virus
Mink coronavirus strain WD1127
Coronavirus AcCoV−JC34
Ferret coronavirus
Rhinolophus bat coronavirus HKU2
NL63−related bat coronavirus
BtRf−AlphaCoV/HuB2013
Miniopterus bat coronavirus HKU8
Beluga whale coronavirus SW1
Betacoronavirus HKU24
Rodent coronavirus
Bat coronavirus
Rousettus bat coronavirus
Bat Hp−betacoronavirus/Zhejiang2013
Bat coronavirus BM48−31/BGR/2008
Wigeon coronavirus HKU20
Magpie−robin coronavirus HKU18
Thrush coronavirus HKU12−600
Bulbul coronavirus HKU11−934
Munia coronavirus HKU13−3514
Common moorhen coronavirus HKU21
Night heron coronavirus HKU19
Sparrow coronavirus HKU17
Porcine coronavirus HKU15
White−eye coronavirus HKU16
Human coronavirus 229E
Bat coronavirus CDPHE15/USA/2006
Porcine epidemic diarrhea virus
Canada goose coronavirus
Infectious bronchitis virus
Duck coronavirus
Turkey coronavirus
Rabbit coronavirus HKU14
Murine hepatitis virus
Human coronavirus OC43
Rat coronavirus Parker
Bovine coronavirus
Human coronavirus HKU1
SARS coronavirus Tor2
Severe acute respiratory syndrome coronavirus 2
Rousettus bat coronavirus HKU9
Betacoronavirus England 1
Middle East respiratory syndrome−related coronavirus
Betacoronavirus Erinaceus/VMC/DEU/2012
Pipistrellus bat coronavirus HKU5
Tylonycteris bat coronavirus HKU4
B
Psittacine adenovirus 1
Psittacine adenovirus 3
Lizard adenovirus 2
Odocoileus adenovirus 1
Bovine adenovirus 6
Bovine atadenovirus D
Fowl aviadenovirus E
Fowl aviadenovirus C
Turkey adenovirus 1
Turkey aviadenovirus 5
Fowl aviadenovirus 5
Fowl aviadenovirus 6
Turkey aviadenovirus 4
Psittacine aviadenovirus B
Pigeon adenovirus 1
Pigeon adenovirus 2
Duck adenovirus 2
Goose adenovirus 4
Simian adenovirus 13
Simian adenovirus DM−2014
Human mastadenovirus D
Human mastadenovirus B
Human mastadenovirus C
Human mastadenovirus F
Baboon adenovirus 3
Simian adenovirus 19
Chimpanzee adenovirus Y25
Cynomolgus adenovirus 1
Simian adenovirus 49
Simian adenovirus 16
Simian adenovirus 8
Human mastadenovirus A
Simian mastadenovirus WIV19
Bat mastadenovirus WIV9
Bat mastadenovirus WIV10
Bat mastadenovirus WIV11
Murine adenovirus 2
Murine adenovirus 3
Equine adenovirus 2
Ovine adenovirus 8
red squirrel adenovirus 1
Bat mastadenovirus WIV17
Bat mastadenovirus WIV18
Equine adenovirus 1
Bat mastadenovirus WIV12
Deer mastadenovirus B
Bottlenose dolphin adenovirus 1
Canine mastadenovirus A
bat adenovirus 3
Skunk adenovirus PB1
Bat adenovirus 2
Bat mastadenovirus G

Fig. S8. A,B Dendrogram visualization of by the shared structure similarity across all members of *Coronaviridae* (A) and *Adenoviridae* (B).

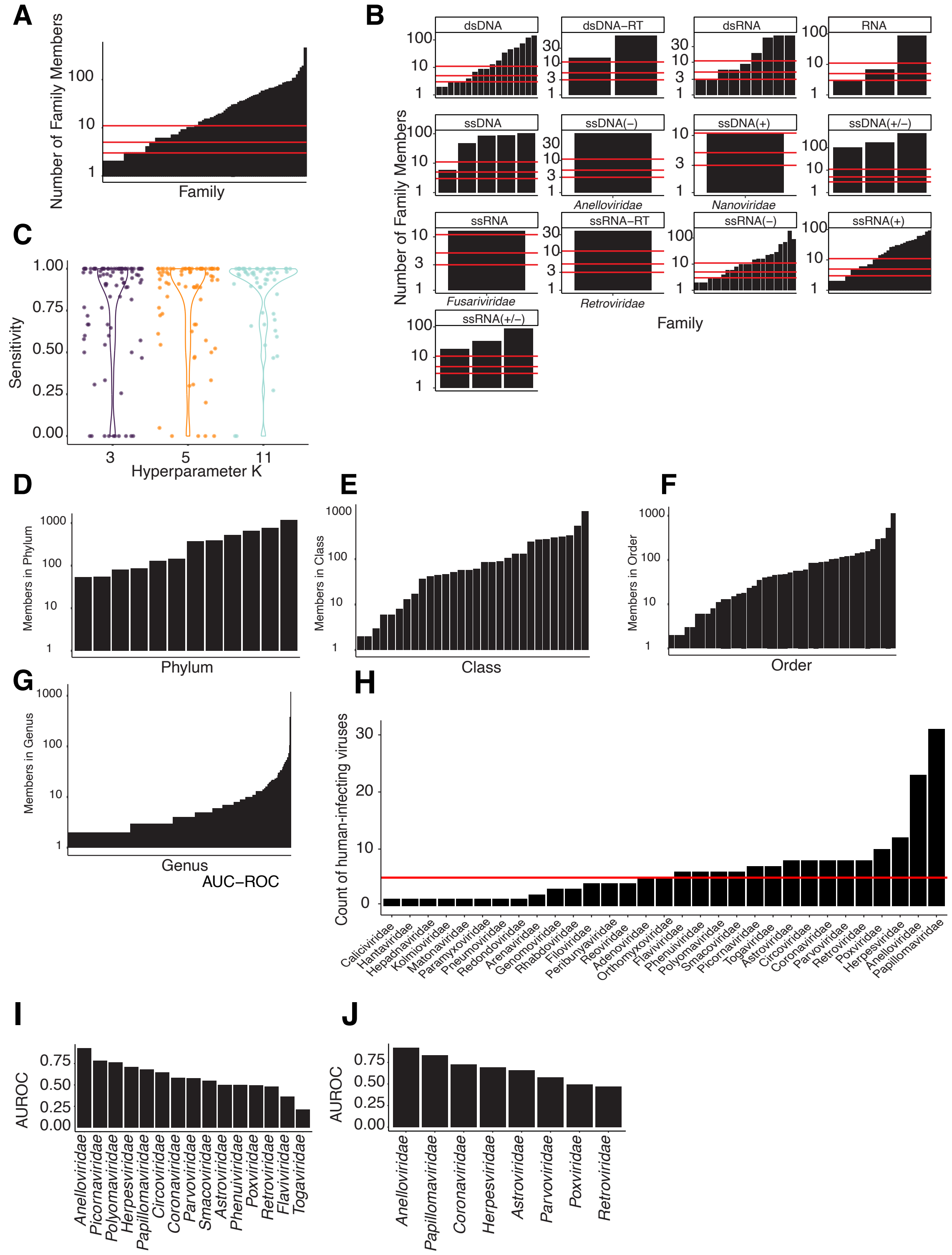

Fig. S9. A Barplot showing the number of members in each viral family, sorted in an ascending order. Red line indicates hyperparameters selected with k = 3,5, 11. **B** Barplot of the number of members in each viral family grouped by molecule type. Red line indicates hyperparameter selection of k (3,5,11). Family names

are indicated if there is only one family represented for the molecule type. **C** Violin plot of sensitivity in viral family classification (y-axis) across different hyperparameters k (x-axis). Each point is a viral family. **D,E,F,G** Barplot of the number of members in each taxon sorted in an ascending order, across different taxonomic levels (x-axes). **H** Barplot showing the number of human-trophic viruses within each viral family. Red line indicates the median number of human-trophic viruses across all families. **I,J** KNN model performance (AUROC) when classifying human vs non-human infecting viruses within each family. Performance is shown for different values of k hyperparameter, k=3 (**I**) and k=7 (**J**).

Supplemental Datasets Legends

Dataset S1 (separate file). Average classification sensitivity obtained using viral structural encodings to classify taxonomy within each taxonomic level

Dataset S2 (separate file). Performance for taxonomy classification using KNN models trained based on amino acid similarity and viral structural encodings, for prediction within each phylum

Dataset S3 (separate file). Putative E4 frames and their respective amino acid sequences in RefSeq *Gammapapillomavirus* Genomes

Dataset S4 (separate file). Nucleotide sequence accessions of viruses in the database included throughout the study, with corresponding species label

Dataset S5 (separate file). Protein Accessions for 67,715 proteins included in the database throughout this study

Supplementary References

Xu S, Dai Z, Guo P, Fu X, Liu S, Zhou L, Tang W, Feng T, Chen M, Zhan L, et al. 2021. ggtreeExtra: Compact Visualization of Richly Annotated Phylogenetic Data. *Mol Biol Evol* 38:4039–4042.

Xu S, Li L, Luo X, Chen M, Tang W, Zhan L, Dai Z, Lam TT, Guan Y, Yu G. 2022. Ggtree: A serialized data object for visualization of a phylogenetic tree and annotation data. *iMeta* 1:e56.
